# Supplementary material for: miR-23b and miR-218 silencing increase Muscleblind-like expression and alleviate myotonic dystrophy phenotypes in mammalian models
Source: Nat Commun. 2018 Jun 26;9:2482. doi: 10.1038/s41467-018-04892-4 (PMC6018771; doi:10.1038/s41467-018-04892-4)
Supplement: Supplementary file 1 — Supplementary Information [file 41467_2018_4892_MOESM1_ESM.pdf]

miR-23b and _miR-218_ silencing increase Muscleblind-like expression and alleviate myotonic dystrophy phenotypes in mammalian models

Cerro-Herreros et al.

Supplementary information

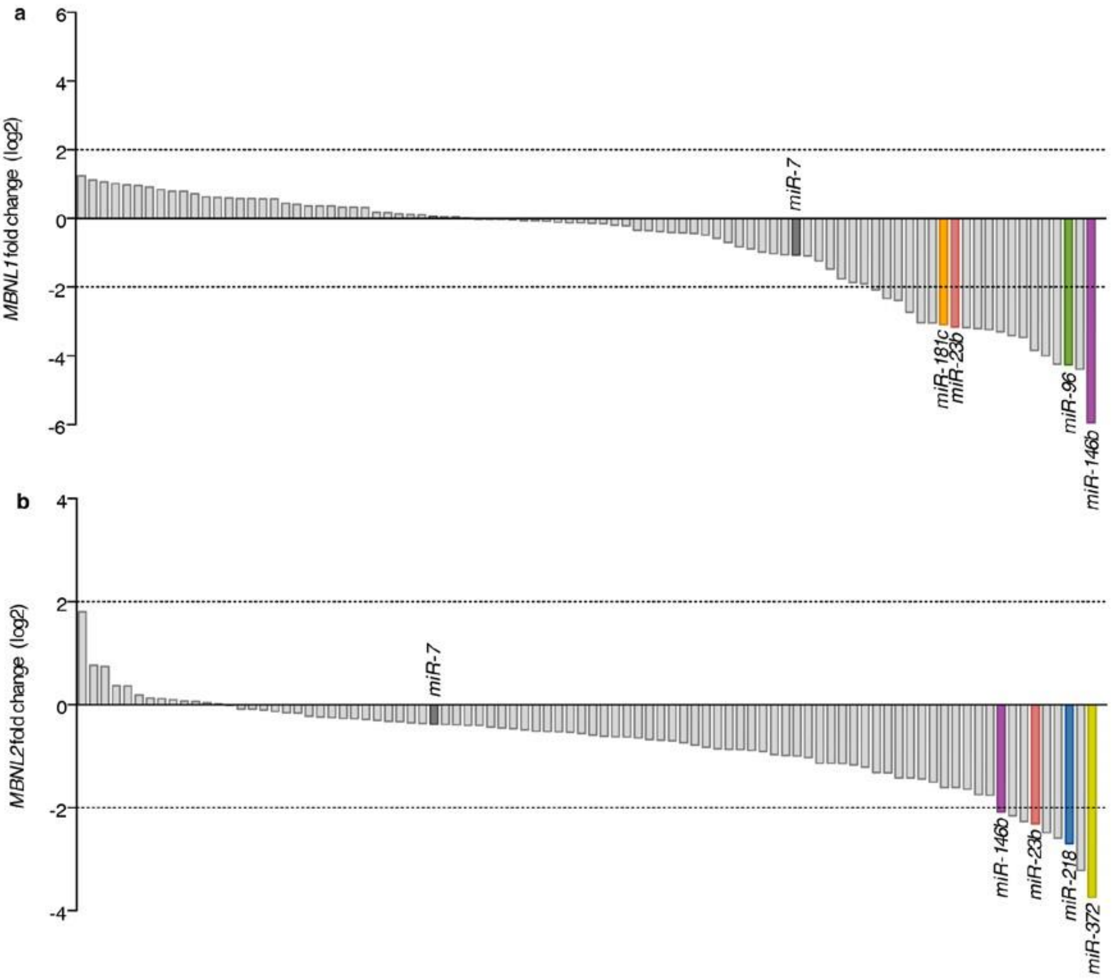

Supplementary Figure 1 Screening of potential miRNAs that repress *MBNL1* and *MBNL2*. (**a, b**) qRT-PCR analyses of *MBNL1* (**a**) and *MBNL2* (**b**) mRNA expression relative to *GAPDH* gene in HeLa cells treated with different miRNA mimics. *miR-372* repressed expression of *MBNL2* and served as positive control for the screen because it was previously shown to regulate this protein[60]. A threshold of 4X fold change was used to select miRNA candidates for validation.

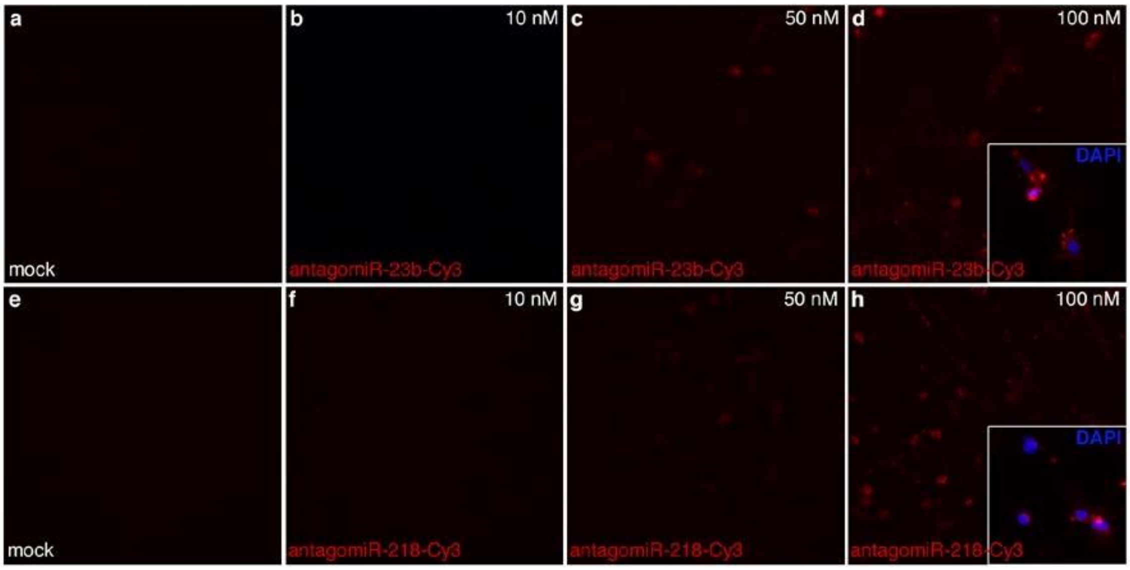

Supplementary Figure 2 Confirmation of cell uptake of antagomiR-23b and -218. Fluorescence images of antagomiR-23b (**b-d**) and antagomiR-218 (**f-h**) labelled with Cy3 (red) at concentrations 10 nM (**b, f**), 50 nM **(c, g)**, and 100 nM (**d, h**) in control cells 48 h after transfection. Untransfected cells (mock) (**a, e**) were used as controls. Nuclei were counterstained with DAPI (blue).

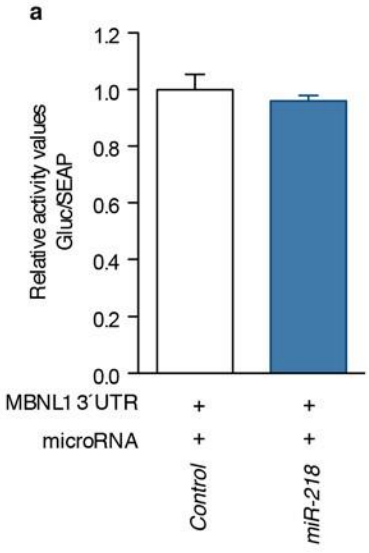

Supplementary Figure 3 *miR-218* does not bind *MBNL1* 3'UTR. miRNA luciferase reporter assay in HeLa cells co-transfected with *MBNL1* 3' UTR fused to Gaussia luciferase and *miR-218* expressing plasmids (*n*= 4). Data are mean ± SEM.

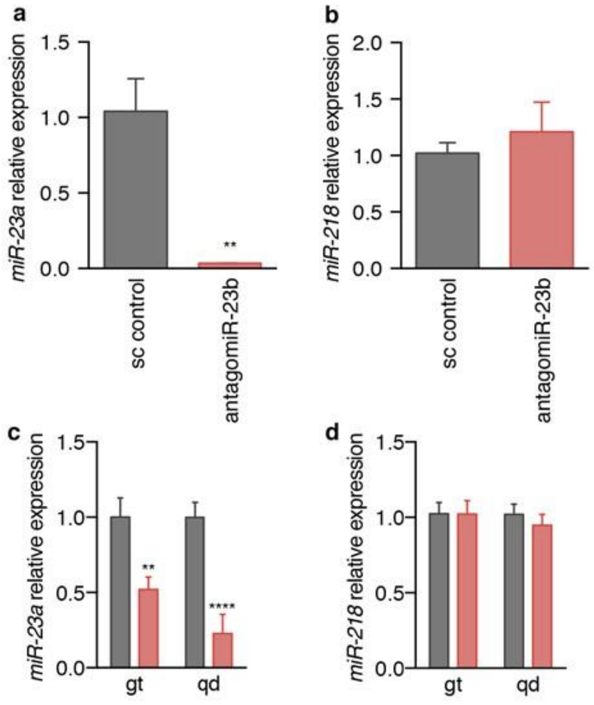

Supplementary Figure 4 AntagomiR-23b silences *miR-23a* but not *miR-218*. qRT-PCR quantification of *miR-23a* (**a**, **c**) and *miR-218* (**b, d**) expression levels in DM1 myoblasts (**a**, **b**) and HSA^{LR} mice (**c**, **d**) treated with antagomiR-23b. Expression levels relative to the endogenous *Gapdh* were normalized to the levels in cells or mice treated with scrambled control antagomiR. The data were analyzed by unpaired Student's *t* test $**p < 0.01$, $****p < 0.001$. Data are mean ± SEM.

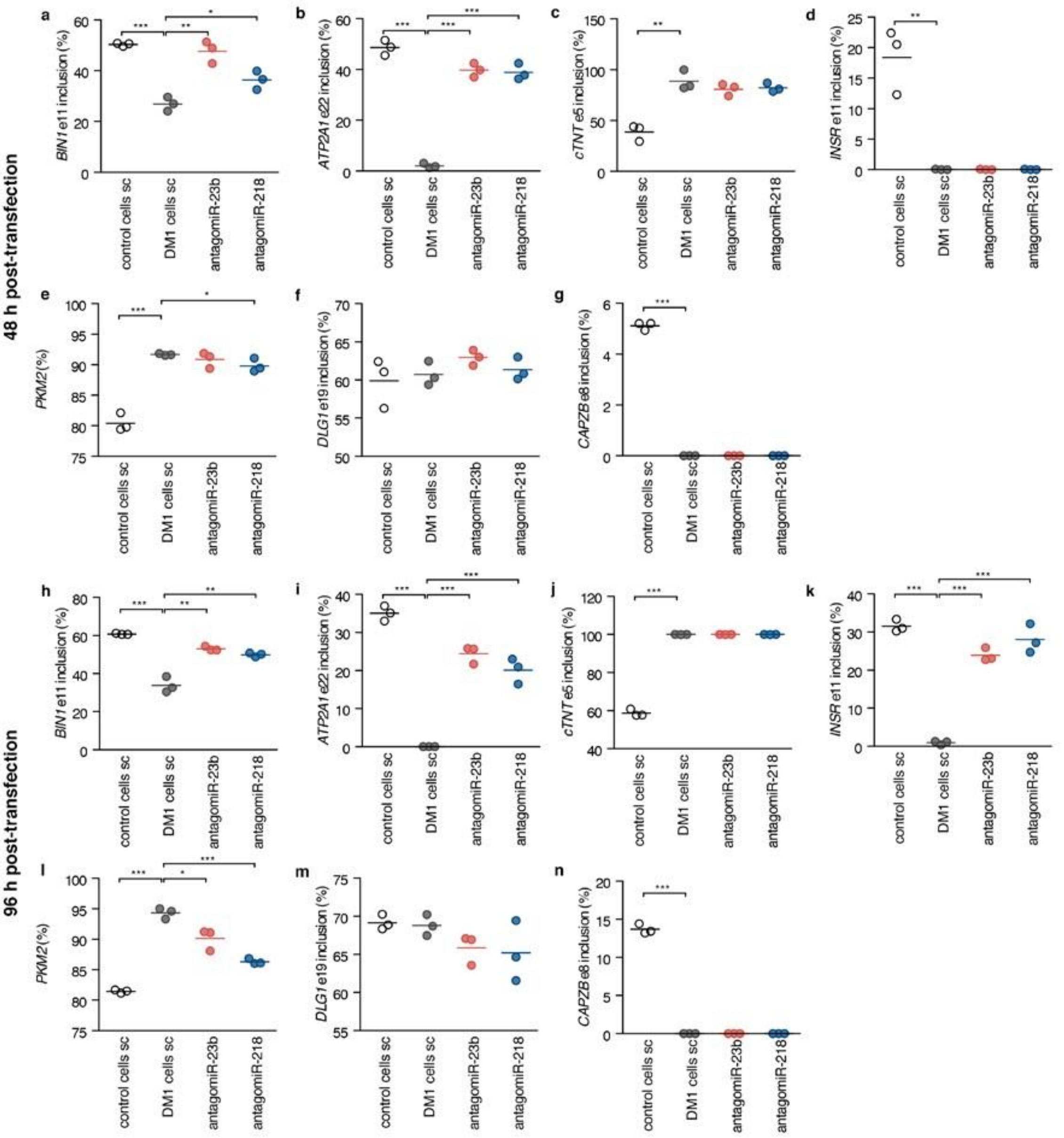

Supplementary Figure 5 RT-PCR quantification of splicing events altered in DM1 cells 48 h and 96 h post-transfection with antagomiRs against *miR-23b* or *miR-218*. Graphs show the percentage (%) of exon inclusion for each gene: *BIN1* exon 11 (**a, h**), *ATP2A1* exon 22 (**b, i**), *cTNT* exon 5 (**c, j**), *INR* exon 11 (**d, k**), *DLG1* exon 9 (**f, m**) and *CAPZB* exon 8 (**g, n**) 48 h (**a-g**) and 96 h (**h-n**) after treatment of DM1 myoblast with the indicated antagomiR. Healthy controls and DM1 myoblasts without treatment are represented too. In the case of *PKM*, bars represent the percentage (%) of PKM2 isoform relative to total *PKM* (*PKM1* + *PKM2*) (**e, l**). Data are mean ± SEM * $p<0.05$, ** $p<0.01$, ***$p<0.001$ in Student's *t* test ($n=3$).

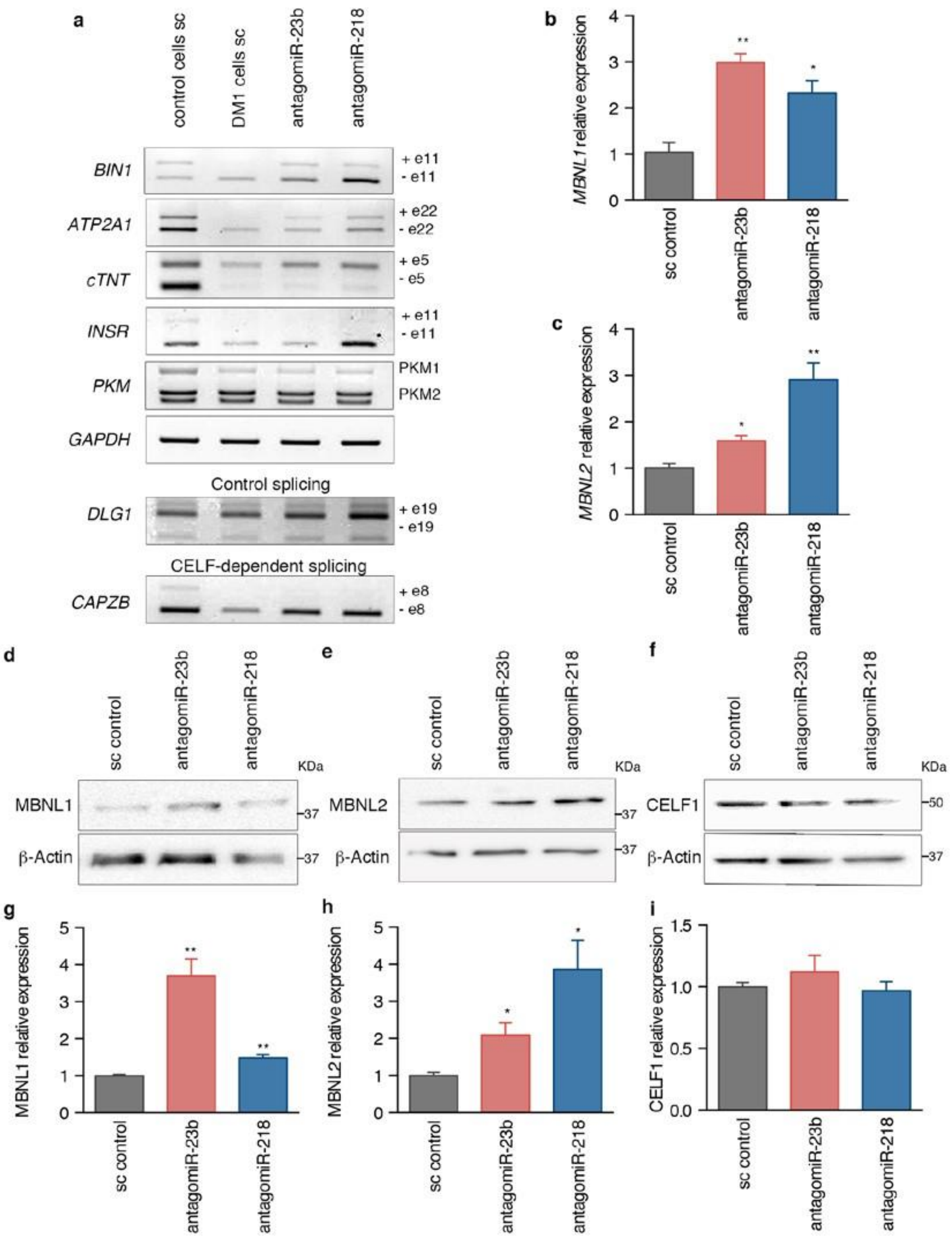

Supplementary Figure 6 Silencing of *miR-23b* or *miR-218* with antagomiRs rescues molecular alterations in DM1 myoblast 48 h post-transfection. **(a)** Semiquantitative RT-PCR analyses of splicing events altered in DM1 cells (*BIN1* exon 11, *ATP2A1* exon 22, *cTNT* exon 5, *INSR* exon 11 and *PKM* isoforms). *GAPDH* was analyzed as internal control. As additional controls, a splicing event not altered in DM1 (*DLG1* exon 9) and CELF1-dependent splicing event (*CAPZB* exon 8) were analyzed. The assays were performed in healthy control myoblast without treatment (Control cells) and DM1 myoblast 48 h after transfection with 50 nM antagomiR-23b, 200 nM antagomiR-218, or scrambled antagomiR (sc) as control. (**b, c**) qRT-PCR analyses of *MBNL1* (**b**)

and *MBNL2* (**c**) expression relative to *GAPDH* and *ACTB* genes in human DM1 myoblasts transfected with the indicated antagomiRs or scrambled control antagomiR (sc). (**d- i**) Representative western blot images and quantification of MBNL1 (**d, g**), MBNL2 (**e, h**) and CELF1 (**f, i**) expression levels in human DM1 myoblasts transfected with the indicated antagomiRs or scrambled control antagomiR (sc). β-ACTIN expression was used as endogenous control. (*n*=3). Data are mean ± SEM. *$p<0.05$, **$p<0.01$, ***$p<0.001$ in Student's *t* test.

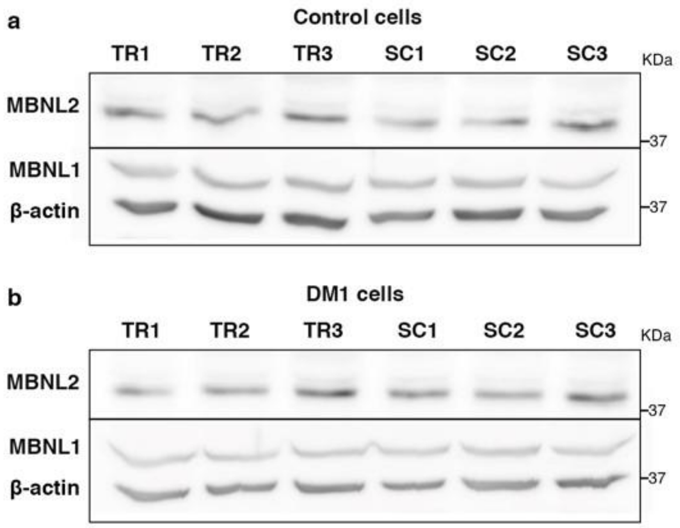

Supplementary Figure 7 Scrambled antagomiR did not alter MBNL1 and MBNL2 levels. Western blot to detect MBNL1 and MBNL2 proteins in control cells (**a**) and DM1 cells (**b**) treated with scrambled antagomiR (SC) or mock-transfected (TR). β-ACTIN was detected as loading control.

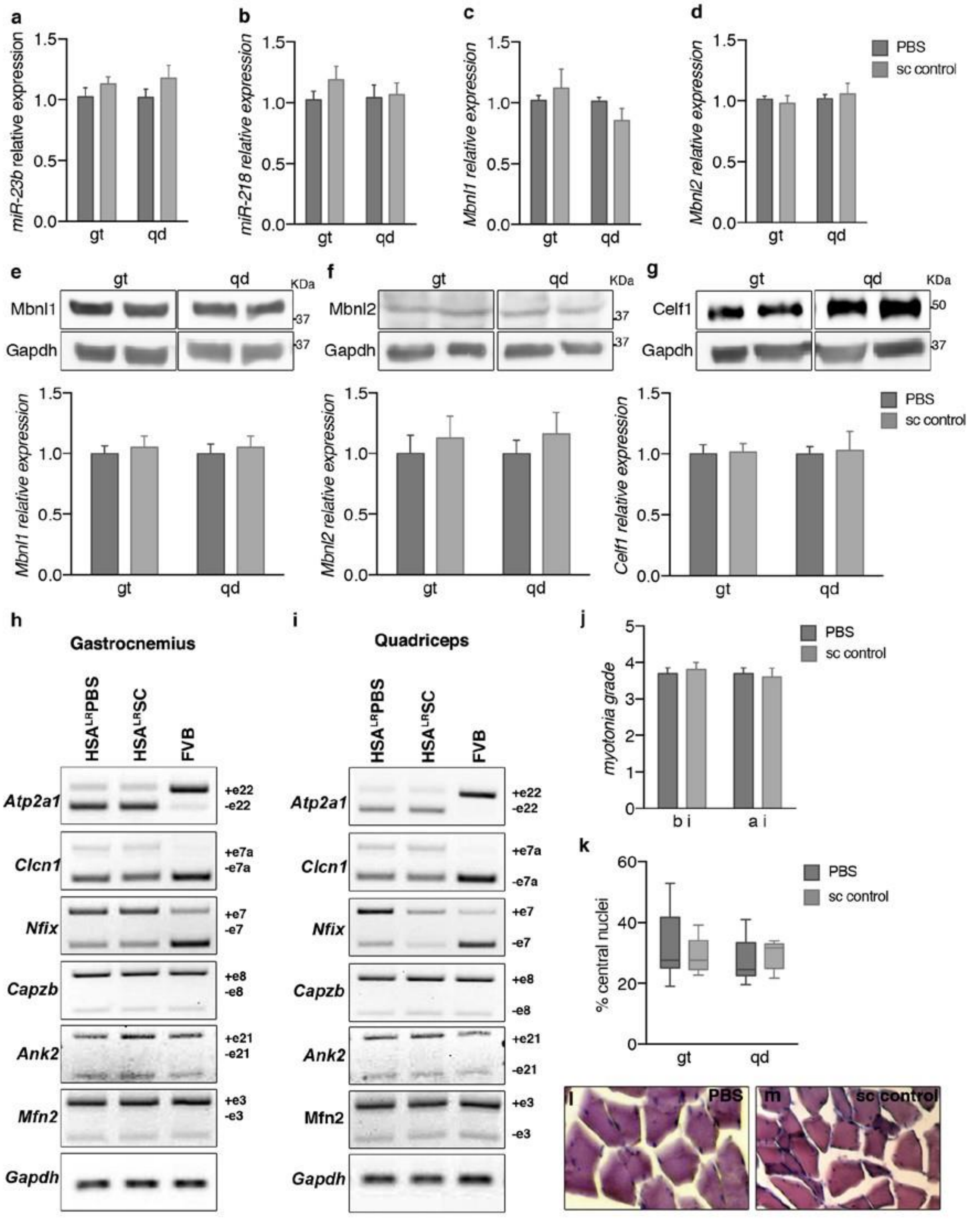

Supplementary Figure 8 Subcutaneous injection of scrambled antagomiR in HSA^{LR} mice control did not reduced *miR-23b* and *miR-218* levels nor increased Mbnl1 and Mbnl2. qPCR

quantification of *miR-23b* and *miR-218* (**a**-**b**) and *Mbnl1* and *Mbnl2* expression levels (**c**-**d**) quadriceps (qd) and gastrocnemius (gt) muscles. (**e**-**g**) Western blotting analysis of Mbnl1 (**e**), Mbnl2 (**f**), and Celf1 (**g**) proteins in mouse gt and qd muscles. (**h-i**) RT-PCR analyses of the splicing of *Atp2a1* exon 22, *Clcn1* exon 7a, *Nfix* exon 7, *Capzb* exon 8, *Ank2* exon 21 and *Mnf2* exon 3 in gastrocnemius (gt) (**a**) and quadriceps (qd) (**b**) muscles. (**j**) Electromyographic myotonia grade in scrambled antagomiR or PBS-treated HSA^{LR} mice before (bi) and four days after injection (ai). Data are mean ± SEM. (**k**) Quantification of the percentage of muscle fibers with central nuclei in gt and qd muscles of PBS or scrambled antagomiR-treated HSA^{LR} mice. (**e-h**) Representative hematoxylin and eosin staining of gt muscles. HSA^{LR} PBS (*n*=10), HSA^{LR} scrambled antagomiR (*n*=5). Data are media ± SEM.

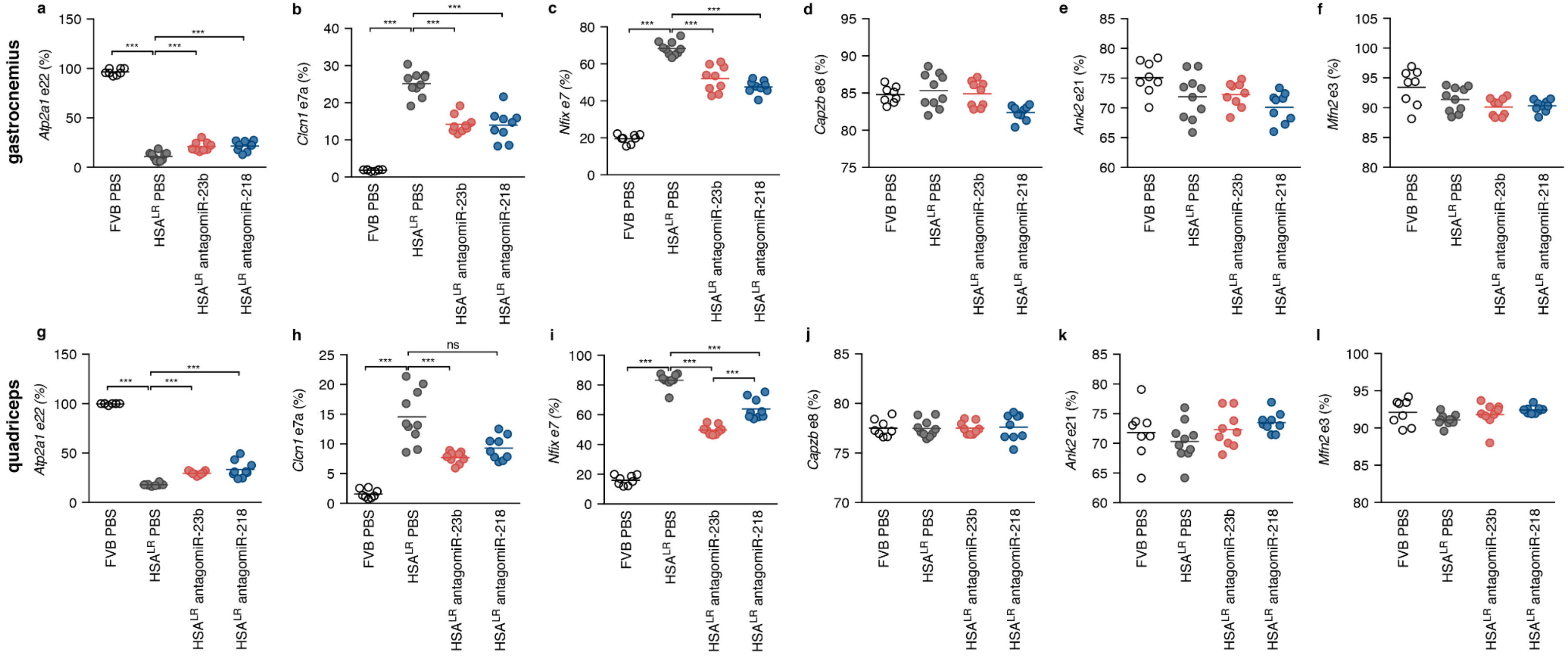

Supplementary Figure 9 Quantification of PSI from *Atp2a1* exon 22, *Clcn1* exon 7a, *Nfix* exon 7, and *Capzb* exon 8 in gastrocnemius (gt) (**a-d**) and quadriceps (qd) (**e-h**) muscles. Endogenous *Gadph* values were used for normalization. Data are media ± SEM. *$p < 0.05$, **$p < 0.01$, ***$p < 0.001$ (Student's t test); FVB (n=8), HSA^{LR} PBS (n=10), HSA^{LR} antagomiR-23b (n=9) and HSA^{LR} antagomiR-218 (n=9).

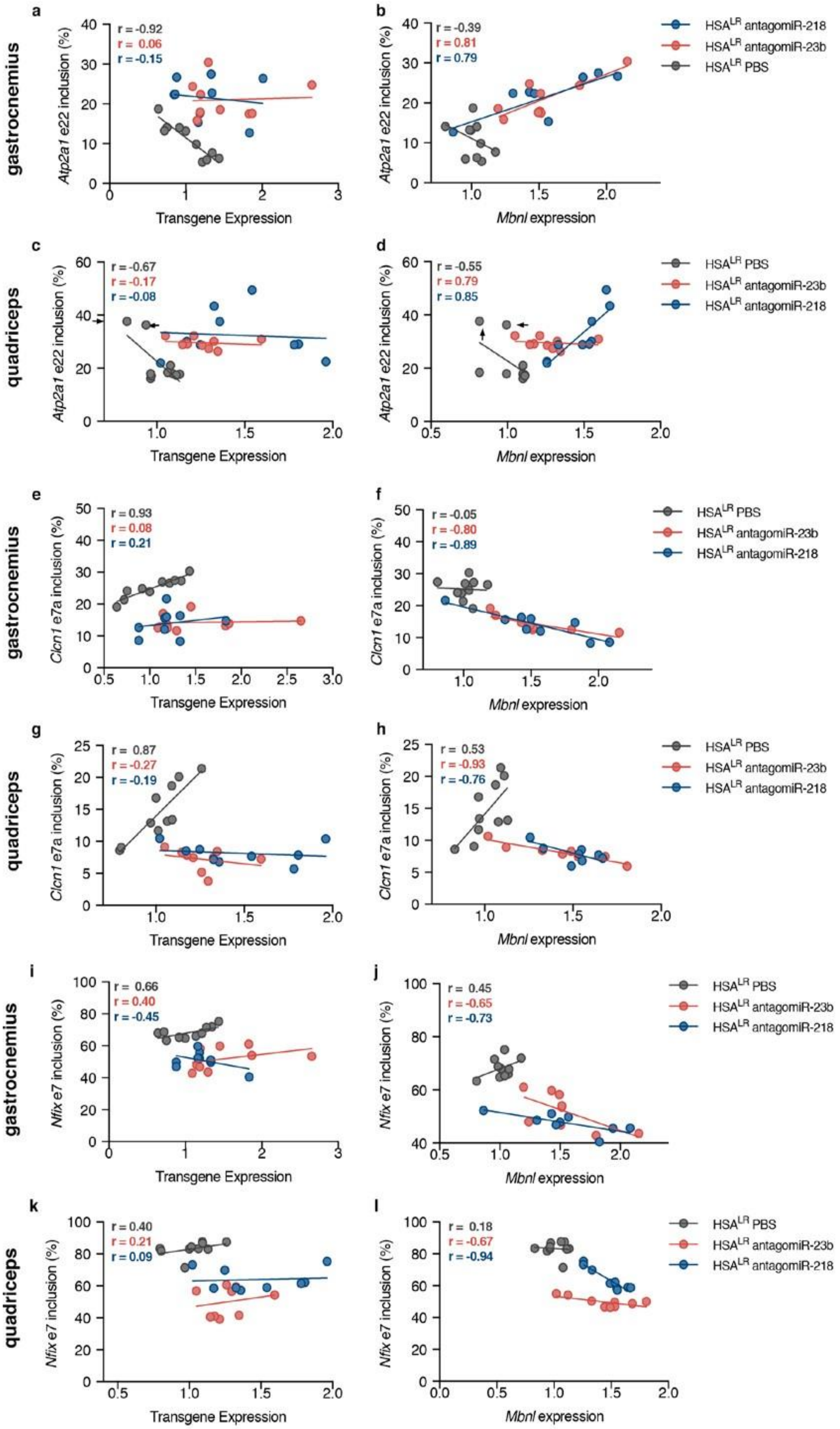
a
gastrocnemius
r = -0.92
r = 0.06
r = -0.15
Atp2a1 e22 inclusion (%)
Transgene Expression
b
r = -0.39
r = 0.81
r = 0.79
Atp2a1 e22 inclusion (%)
Mbnl expression
HSA^LR antagomiR-218
HSA^LR antagomiR-23b
HSA^LR PBS
c
quadriceps
r = -0.67
r = -0.17
r = -0.08
Atp2a1 e22 inclusion (%)
Transgene Expression
d
r = -0.55
r = 0.79
r = 0.85
Atp2a1 e22 inclusion (%)
Mbnl expression
HSA^LR PBS
HSA^LR antagomiR-23b
HSA^LR antagomiR-218
e
gastrocnemius
r = 0.93
r = 0.08
r = 0.21
Clcn1 e7a inclusion (%)
Transgene Expression
f
r = -0.05
r = -0.80
r = -0.89
Clcn1 e7a inclusion (%)
Mbnl expression
HSA^LR PBS
HSA^LR antagomiR-23b
HSA^LR antagomiR-218
g
quadriceps
r = 0.87
r = -0.27
r = -0.19
Clcn1 e7a inclusion (%)
Transgene Expression
h
r = 0.53
r = -0.93
r = -0.76
Clcn1 e7a inclusion (%)
Mbnl expression
HSA^LR PBS
HSA^LR antagomiR-23b
HSA^LR antagomiR-218
i
gastrocnemius
r = 0.66
r = 0.40
r = -0.45
Nfix e7 inclusion (%)
Transgene Expression
j
r = 0.45
r = -0.65
r = -0.73
Nfix e7 inclusion (%)
Mbnl expression
HSA^LR PBS
HSA^LR antagomiR-23b
HSA^LR antagomiR-218
k
quadriceps
r = 0.40
r = 0.21
r = 0.09
Nfix e7 inclusion (%)
Transgene Expression
l
r = 0.18
r = -0.67
r = -0.94
Nfix e7 inclusion (%)
Mbnl expression
HSA^LR PBS
HSA^LR antagomiR-23b
HSA^LR antagomiR-218

Supplementary Figure 10 Pearson`s correlations between *Atp2a1* e22 (**a-d**), *Nfix* e7 (**e-h**) or *Clcn1* exon 7a (**i-l**) exon inclusion and HSA^{LR} transgene expression (**a, c, e, g, i, k**) or *Mbnl* expression (**b, d, f, h, j, l**). The best correlation was observed for *Atp2a1* PSI, suggesting that this splicing event strongly depends on repeats toxicity. In **c** and **d**, arrows point to weakly affected DM1 mice, which have less transgene expression. HSA^{LR} PBS (*n*=10), HSA^{LR} antagomiR-23b (*n*=9). HSA^{LR} antagomiR-218 (*n*=9).

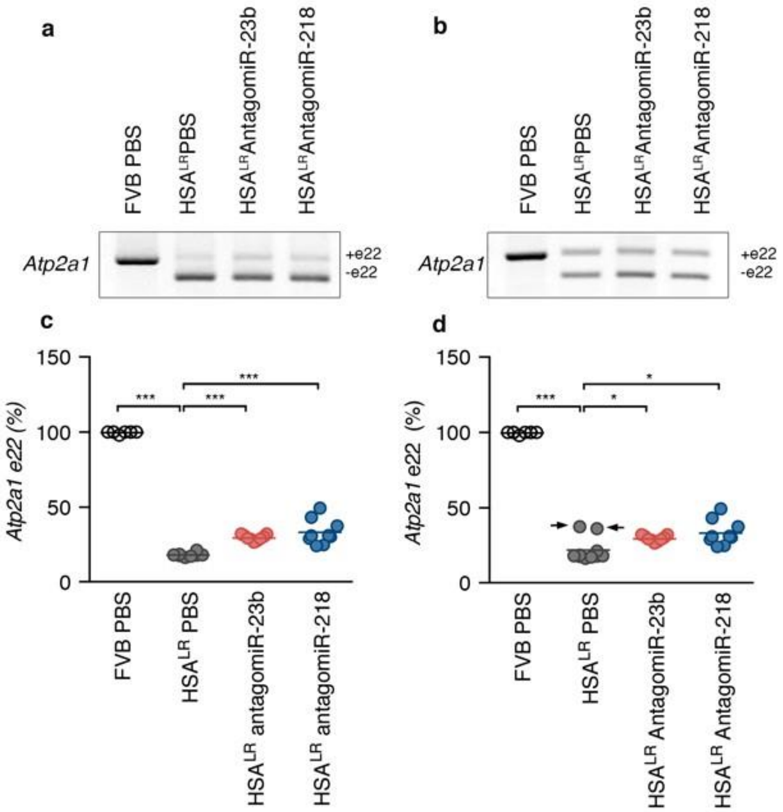

Supplementary Figure 11 Splicing of *Atp2a1* exon 22 variability in HSA^{LR} PBS treated control group. (**a-b**) Representative RT-PCR determinations of the splicing of *Atp2a1* exon 22 and their quantification (**c-d**). Note the difference in the quantification using the three more severely affected PBS-treated mice (**a, c**) or the complete group (**b, d**), including the less affected mice (arrows, **d**). Data are mean ± SEM. $p* < 0.05$, $**p < 0.01$, $***p < 0.001$ (Student's *t* test); FVB (*n*=8), HSA^{LR} PBS (*n*=8 in **c** and *n*=10 in **d**), HSA^{LR} antagomiR-23b (*n*=9) and HSA^{LR} antagomiR-218 (*n*=9).

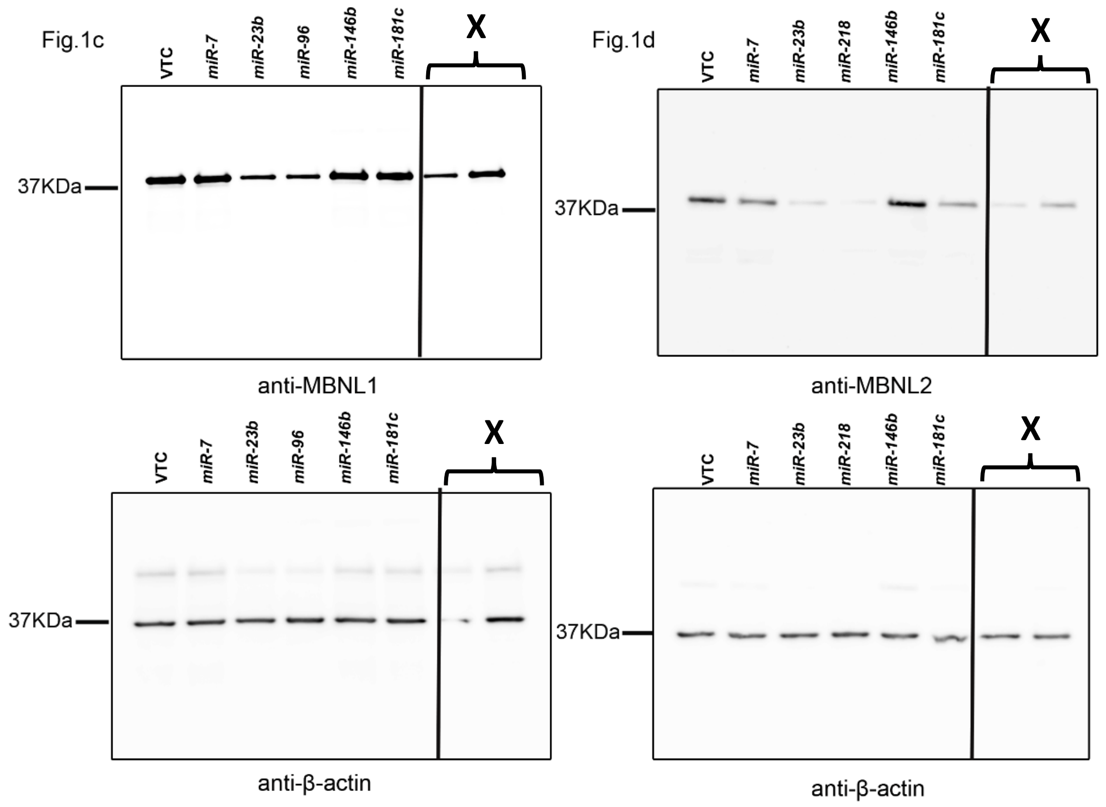

Supplementary Figure 12 Uncropped western blots for Figure 1. Crosses mark membrane lanes from unrelated experiments.

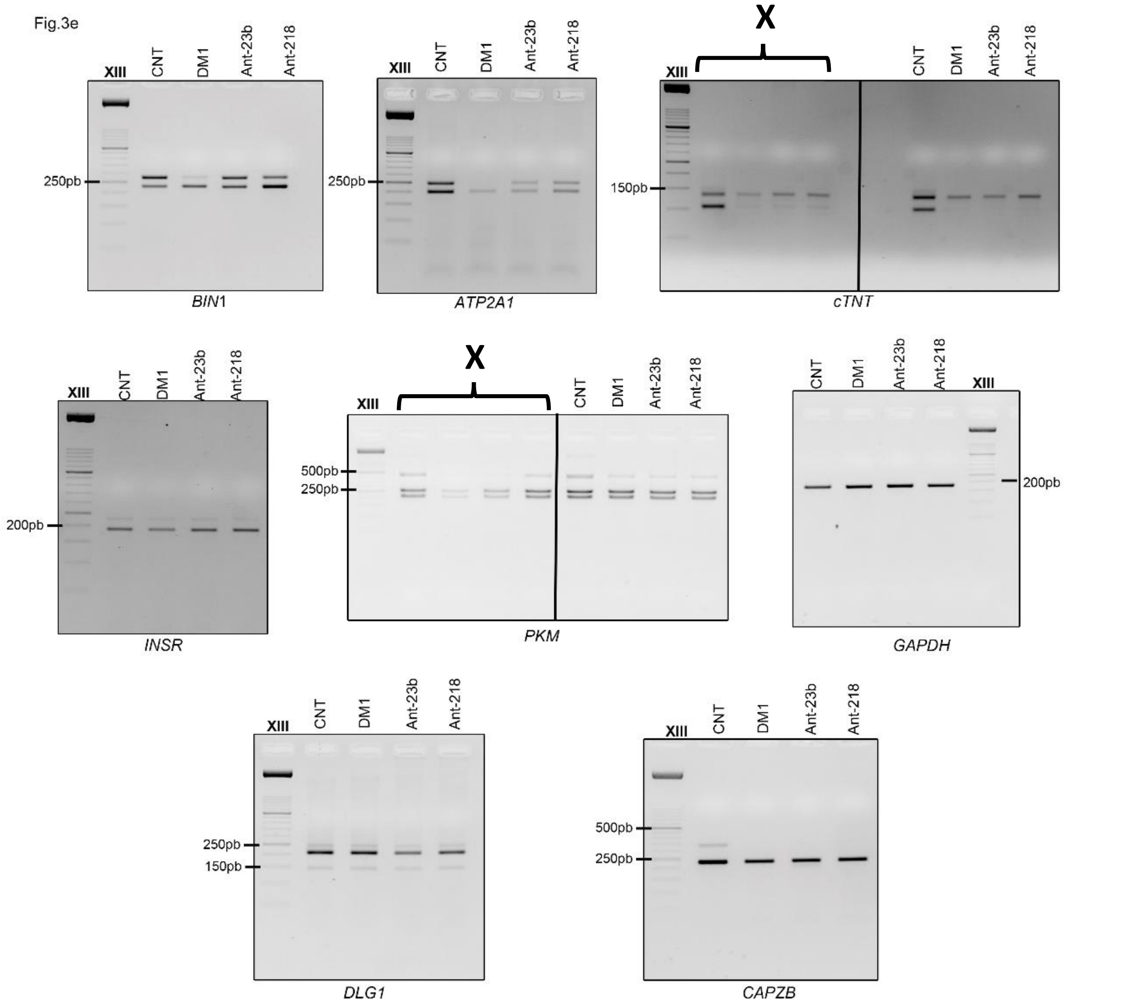

Supplementary Figure 13 Uncropped agarose gels for figure 3. Crosses mark agarose gel lanes from unrelated experiments.

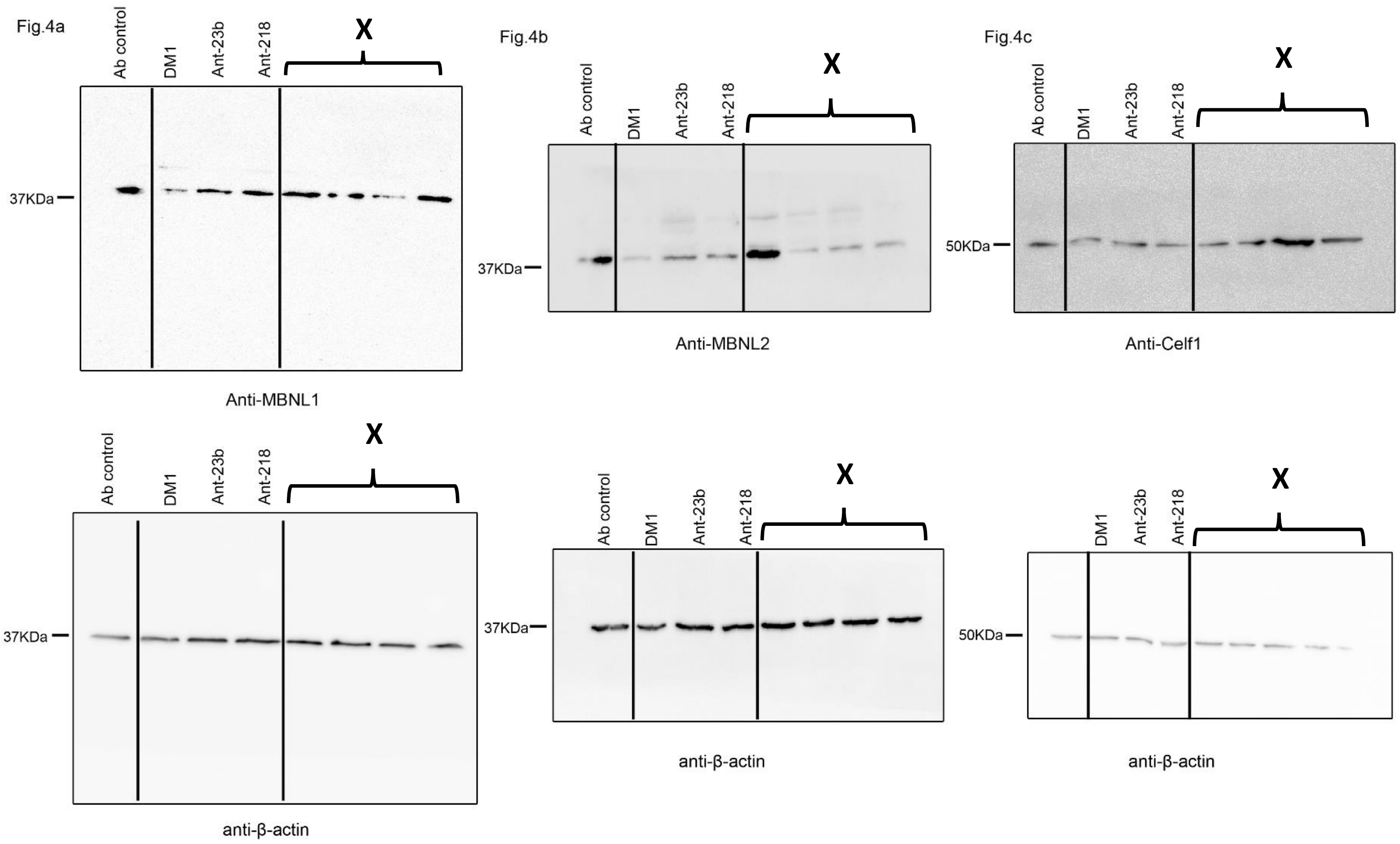

Supplementary Figure 14 Uncropped western blots for Figure 4. Crosses mark membrane lanes from unrelated experiments. “Ab control” designates an internal control of antibody specificity.

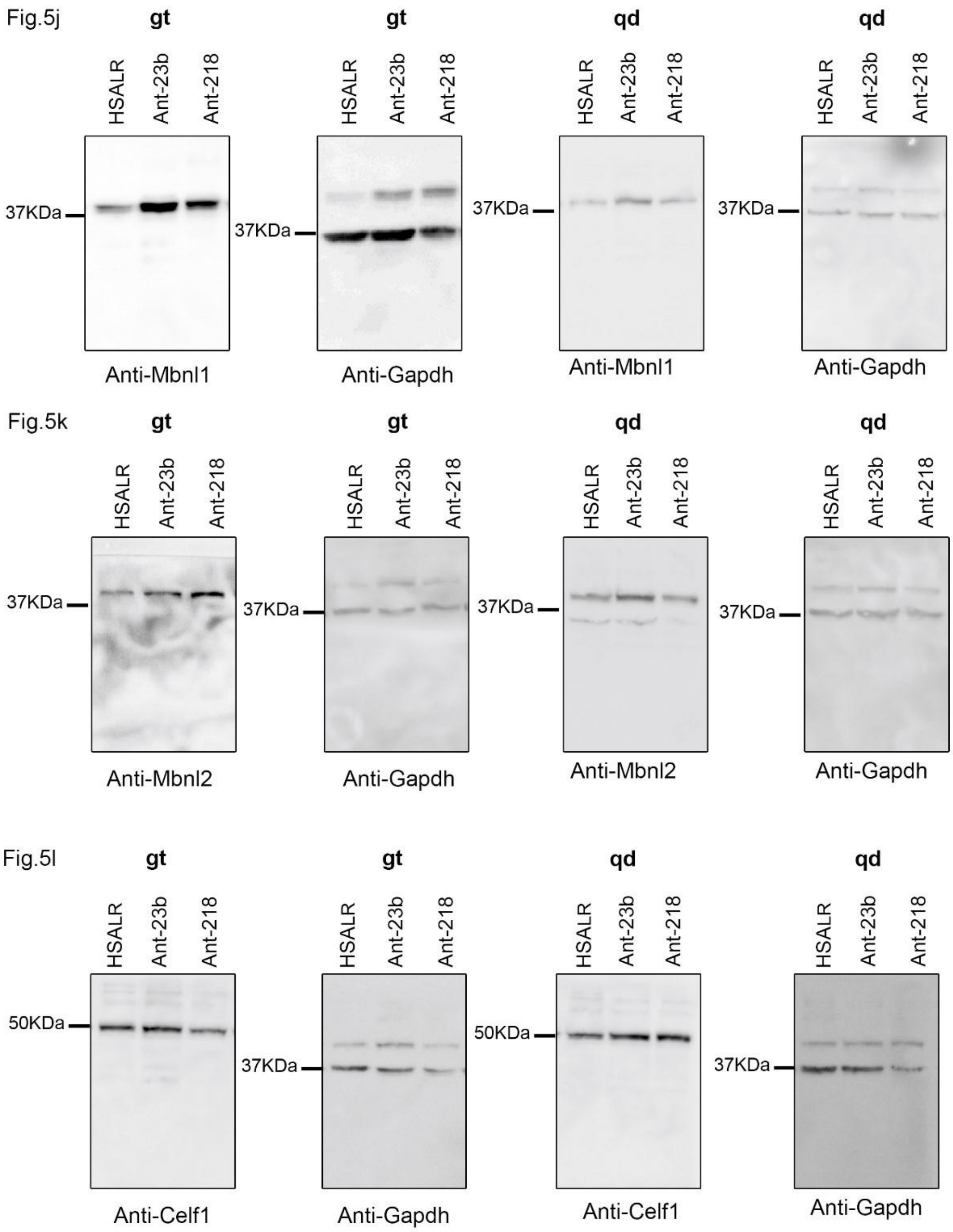

Supplementary Figure 15 Uncropped western blots for Figure 5.

Fig.6a-b

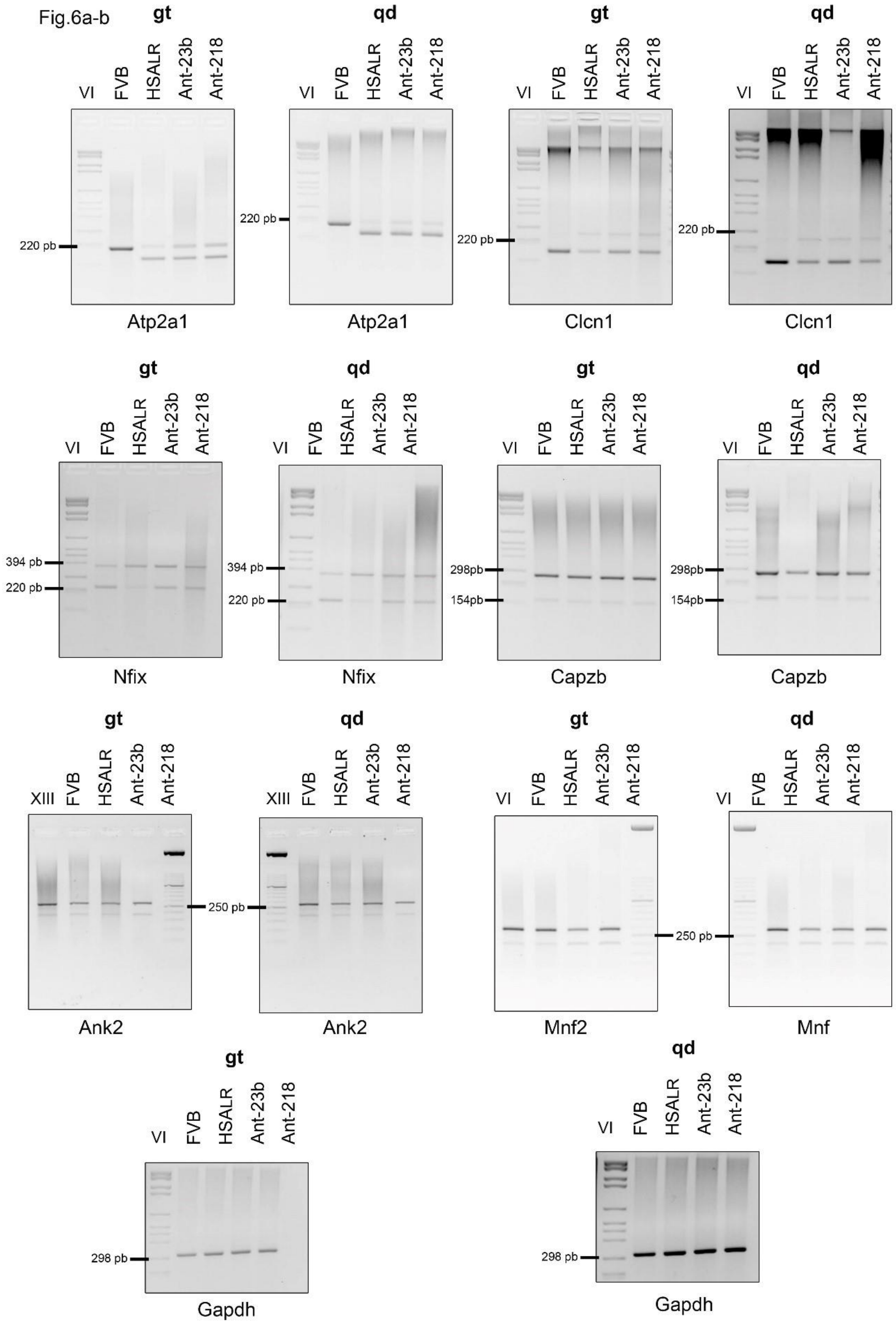

gt
qd
VI
FVB
HSALR
Ant-23b
Ant-218
220 pb
Atp2a1
Atp2a1
Clcn1
Clcn1
394 pb
220 pb
298pb
154pb
Nfix
Nfix
Capzb
Capzb
XIII
250 pb
Ank2
Ank2
Mnf2
Mnf
298 pb
Gapdh
Gapdh

Supplementary Figure 16 Uncropped agarose gels for Figure 6.

Gene	***miR***	***1***	***2***	***3***	***4***	***5***	***6***	***7***	***8***	***9***	***Number of predictions***
MBNL1	*miR-23b*	+	+	+	+	+	+	+	-	+	*8*
	miR-96	+	+	+	+	-	+	+	+	+	*8*
	miR-146b	-	+	-	-	-	+	+	+	-	*4*
	miR-181c	+	+	+	+	-	+	+	+	+	*8*
MBNL2	*miR-23b*	+	+	-	-	-	+	+	-	-	*4*
	miR-146b	-	+	-	-	-	+	+	+	-	*4*
	miR-218	+	+	+	-	+	+	+	+	+	*8*
	miR-372	+	+	-	-	+	+	+	+	+	*7*

Supplementary Table 1 Prediction of binding of candidate miRNAs to the *MBNL1* or *MBNL2* 3'UTR sequences according to the indicated programs. Data were taken from miRecords and miRDIP databases. + positive prediction; - negative prediction. **1**: MicroT; **2**: MiRanda; **3**: MirTar2_V4.0; **4**: Mir Target2; **5**: Pic Tar; **6**: PITA; **7**: RNA hybrid; **8**: RNA22; **9**: TargetScan.

Primer	Sequence(5'→3')	Exon
GAPDH Fwd	CATCTTCCAGGAGCGAGATC	-
GAPDH Rv	GTTCACACCCATGACGAACAT	
ATP2A Fwd	GATGATCTTCAAGCTCCGGGC	22
ATP2A Rv	CAGCTCTGCCTGAAGATGTG	
BIN1 Fwd	CTCAACCAGAACCTCAATGATGTG	11
BIN1 Rv	CTGAGATGGGGACTTGGGGAG	
INSR Fwd	TGCTGCTCCTGTCCAAAGAC	11
INSR Rv	GAAGTGTTGGGGAAAGCTG	
PKM Fwd	CTGAAGGCAGTGATGTCGCC	*
PKM Rv	ACCCGGAGGTCCACGTCTC	
CAPZB Fwd	GGAGAAGGATGAAACTGTGAGTG	8
CAPZB Rv	CAGAGGTTTAGCATTGCTGCT	8
DLG1 Fwd	AGCCCGATTAAAAAACAGTGA	19
DLG1 Rv	CGTATTCTTCTTCGACCACGGT	
cTNT Fwd	ATAGAAGAGGTGGTGGAAGAGTAC	5
cTNT Rv	GTCTCAGCCTCTGCTTCAGCATCC	
Gapdh Fwd	ATCAACGGGAAGCCCATCAC	-
Gapdh Rv	CTTCCACAATGCCAAAGTTGT	
Atp2a Fwd	GCTCATGGTCCTCAAGATCTCAC	22
Atp2a Rv	GGGTCAGTGCCTCAGCTTTG	
Clcn1 Fwd	GTCCTCAGCAAGTTTATGTCC	7a
Clcn1 Rv	GAATCCTCGCCAGTAATTCC	
Nfix Fwd	TCGACGACAGTGAGATGGAG	7
Nfix Rv	CAAACTCCTTCAGCGAGTCC	
Capzb Fwd	GCACGCTGAATGAGATCTACTTTG	8
Capzb Rv	CCGGTTAGCGTGAAGCAGAG	

Supplementary Table 2 Sequences of oligonucleotides used for semiquantitative RT-PCR. * Primers used to quantify amounts of *PKM1* and *PKM2* isoforms by real-time PCR.
