# Supplementary material for: miR-23b and miR-218 silencing increase Muscleblind-like expression and alleviate myotonic dystrophy phenotypes in mammalian models
Source: Nat Commun. 2018 Jun 26;9:2482. doi: 10.1038/s41467-018-04892-4 (PMC6018771; doi:10.1038/s41467-018-04892-4)
Supplement: Supplementary file 3 — Description of Additional Supplementary Files [file 41467_2018_4892_MOESM3_ESM.pdf]

**Description of Additional Supplementary Files**

**File Name: Supplementary Data 1**
**Description:** contains the sequences of original and mutated 3'UTR of MBNL1 and MBNL2.

**File Name: Supplementary Data 2**
**Description:** includes blood analyses of mice after 6 weeks of antagomiR injection.
