# Supplementary material for: miR-23b and miR-218 silencing increase Muscleblind-like expression and alleviate myotonic dystrophy phenotypes in mammalian models
Source: Nat Commun. 2018 Jun 26;9:2482. doi: 10.1038/s41467-018-04892-4 (PMC6018771; doi:10.1038/s41467-018-04892-4)
Supplement: Supplementary file 4 — Supplementary Data 1 [file 41467_2018_4892_MOESM4_ESM.pdf]

A) MBNL1

Original sequence (in vector pEZX-MT05):

ccacaagtatgttacccagatgtagaattttcatcactaaacaatcatgctaaagaggaaaggacagtgt
gcttggttagagtaaaggacgaggtcattagccatattgtatatatcgtcaagcaacacacacaaaagtt
cctcagccacaagacatccacatattgcatgttaaccagaagaaaagacaacattttccggaaatccact
gcacactgttgcctatacactttgtacatttaattgatatttgtgctgaggtgatattcctgtctaaaag
aacaacattgtctttcttttctagcacagagttatgcattcaaagatgcatacctagttagtttcctata
tattcatgccatcttgaaaagacagactatggtgtaaccatgattctattatgtattggtacgtctgtag
accaagatataatttttaaaaataagtttatttctttcaaggtttacaaataacaaaggtgcaccttgt
atttaaaattgccattatagatgagagcgtgcatgcacagtcattttgtttaagagtaatatttttaat
gtaatagattgtaagacgtggtgagggagggatctgacagagatgaatgtgccaagcaaaccacaactg
tgtatatttaaagcacatcatggctttaagtaccatgttgttaaggattctcatgaagtgccatagact
gtacatcaaattagagtattatttcttcagtgttattgttttcagagccacatttgttgcatatttgct
agtactaatcagtcaaagggcaccattcttttttttttttttgaaaccaaagctgtctcagaaatggcc
aatttaactttacagtaacaatagacagcacaacacaaactctctcaatacagataaactcacacatact
ggagatatatatataatagatatatataaaattattttaatgcattgtagtgtaatatttatgcatacta
tactgtataacatgttattcaaaagggattgccatttctgagacacagtaacaaaaaaatgaggaaatta
ttttgcttctatttatagcctctgtcaaaagtcaaaagactataaatgctttgcaaaaatggtttcacgt
ttgcttaaatgcttcatcacagtcacattcaaaatagtgactctaaacaaagaagaaagcagcactgtca
tcagatgcatgataaaccaaaatatgaaaatgggaaatgtttaattaacctagtaattgggtgggttaag
tacatgggtgaattttatatgtgattttgttttgttttgttttgttcagattaactgcttatagcctta
gaaagcctttacaaaattaaaaaaaaaaatagatgtgcattcagtttttaagaatggaatcatccaaagg
aattcctttttttgaggtttggatgttgcagctagtaaaggatatttttgctctgttcagcagttctaaa
aattgctgaagtaggggccaggtcactggtagttatagtatggaatgggagaagtgaaagttcagttata
gaactttccatacttccaagtttactgcaagtttttatgcttgagagagatgctttctaatataagactg
atgtgttgattttactgattgtactgtacatctattaaagccttagattattacattacgggttggaacc
cataccaatgtaatttcaatcgtgttaagaaagtaatggtgacttcacatgttattgtagttagttacat
tatagaatattacttatttttcttgttaaaatgtagtttttcatttcctacatttattagattttcattt
tctattaacaattgaataccatttcagtttatagacttgttttattagattttaccaatgaattttcaa
aatacaaaaaaaagtagtttttccttcataacatactcagttttgaattacatgtagtgtcacatgaata
ttcgtattgttaactaaatgatttatatttactgatttaatattacagtgtaagaatgtcagtcattgt
tagttcttgtctagttttcattaaaagaacaaagatcttttatatggatatcttataaatatataatcat
tgctaagtaagaagttaagttgttgctatcgcaacaatcctggcagacaattgagtaatattttgatgat
ttattttgtttgtaattagttattataagaagatctagatcctagatattagaataaaatttattttcta
ctgtatccatttcaaatgttaaaatattgtttaatatttttgaaatccctgagtatcaggccttgttata
aataagctgcataatcaataaatagaacaagggactttttgttgataatccaaatactcaaagtttacgt
aatgaaaattatagcgtgtgtgcaaactcttgagggttgattatgctgcaatttagcatgttggaacgtc
tagggagaaggttgacttttgcacttctgtatatagtcaaaagagagaaacctgtataatagtaagatc
ttattttgaataaaaacgtctataattacaaggagttttgttaaggctaatacaatgacagactgagcaa
aattgcttgcaaaagtggcacagagttagcactccataccccttcaaacatgttgctttgctttcttgtg
gacagcttgtagtttgccaggatttttcagctggaaagatacgccatcctttcaaaccctcatgactga
caaaaactccatggggccaaatctgcctgaagatcattaccaaaaatagcaggtacttctaccattaagg
tgaaatcatggatcagatattccttacattttcaaaactactgcatgtttaaaacttcaacaaaaaaaag
agagaaagaactatactaagaacatatattattcagatcagtttctgccaatttcagtggtttattgttc
acaaaaaaatcttcaaaacaagtattgactttcacaaaatttaaatcataaacaggcaaaccaaacagca
cactgtagctatagttgttatgtgattgtttttaattgctgtaggatcctgttctttcagcaggtgaaa
aataaaacgcagttcaaatttcatggttttaattttcaactcagaagcactcaaaaatgcaaaatgtgat
aatgggcacttgtttaaaagaattagtgtatccagccttcactccagctggttaaaaatgttgcacttat
cagcaaccctaccactttcatctgctgaaaggacaaatgtgcttggttttactattatgtaatcacaact
tactttctgcttgtagttgcttaaaattatgtattttgtcttggctgcaatttgttttatgcttatttt
attattactgcagtagttgactttgctgtatggaaaaataaagtgaaattgccctaataaacttctctt
tcttaagtaaaaaaaaaaaaaaaaaaaaa

DELETIONS:

> MBNL1 23b mut

ccacaagtatgttacccagatgtagaattttcatcactaaacaatcatgctaaagaggaaaggacagtgt
gcttggttagagtaaaggacgaggtcattagccatattgtatatatcgtcaagcaacacacacaaaagtt
cctcagccacaagacatccacatattgcatgttaaccagaagaaaagacaacattttccggaaatccact
gcacactgttgcctatacactttgtacatttaattgatatttgtgctgaggtgatattcctgtctaaaag
aacaacattgtctttcttttctagcacagagttatgcattcaaagatgcatacctagttagtttcctata
tattcatgccatcttgaaaagacagactatggtgtaaccatgattctattatgtattggtacgtctgtag
accaagatataatttttaaaaataagtttatttctttcaaggtttacaaataacaaaggtgcaccttgt
atttaaaattgccattatagatgagagcgtgcatgcacagtcatttttgtttaagagtaatatttttaat
gtaatagattgtaagacgtggtgagggagggatctgacagagatgaatgtgccaagcaaaaccacaactg
tgtatatttaaagcacatcatggctttaagtaccatgttgttaaggattctcatgaagtgccatagact
gtacatcaaattagagtattatttcttcagtgttattgttttcagagccacatttgttgcatatttgct
agtactaatcagtcaaagggcaccattcttttttttttttttgaaaccaaagctgtctcagaaatggcc
aatttaactttacagtaacaatagacagcacaacacaaactctctcaatacagataaactcacacatact
ggagatatatatataatagatatatataaaattatttaatgcattgtagtgtaatatttatgcatacta
tactgtataacatgttattcaaaagggattgccatttctgagacacagtaacaaaaaaatgaggaaatta
ttttgcttctatttatagcctctgtcaaaagtcaaaagactataaatgctttgcaaaaatggtttcacgt
ttgcttaaatgcttcatcacagtcacattcaaaatagtgactctaaacaaagaagaaagcagcactgtca
tcagatgcatgataaaccaaaatatgaaaatgggaaatgtttaattaacctagtaattgggtgggttaag
tacatgggtgaattttatatgtgatttttgttttgttttgttttgttcagattaactgcttatagcctta
gaaagccttttacaaaattaaaaaaaaaatagatgtgcattcagtttttaagaatggaatcatccaaagg
aattcctttttttgaggtttggatgttgcagctagtaaaggatatttttgctctgttcagcagttctaaa
aattgctgaagtaggggccaggtcactggtagttatagtatggaatgggagaagtgaaagttcagttata
gaactttccatacttccaagtttactgcaagttttttatgcttgagagagatgctttctaatataagactg
atgtgttgatttactgattgtactgtacatctattaaagccttagattattacattacgggttggaacc
cataccaatgtaatttcaatcgtgttaagaaagtaatggtgacttcacatgttattgtagttagttacat
tatagaatattacttatttttcttgttaaaatgtagtttttcatttcctacatttattagattttcattt
tctattaacaattgaataccatttcagtttatagacttgttttattagatttaccaatgaatttttcaa
aatacaaaaaaaagtagtttttccttcataacatactcagttttgaattacatgtagtgtcacatgaata
ttcgtattgttaactaaatgatttatatttactgatttaatattacagtgtaagaatgtcagtcattgt
tagttcttgtctagttttcattaaaagaacaaagatcttttatatggatatcttataaatatataatcat
tgctaagtaagaagttaagttgttgctatcgcaacaatcctggcagacaattgagtaatattttgatgat
ttattttgtttgtaattagttattataagaagatctagatcctagatattagaataaaatttatttcta
ctgtatccatttcaaatgttaaaatattgtttaatattttgaaatccctgagtatcaggccttgttata
aataagctgcataatcaataaatagaacaagggactttttgttgataatccaaatactcaaagtttacgt
aatgaaaattatagcgtgtgtgcaaactcttgagggttgattatgctgcaatttagcatgttggaacgtc
tagggagaaggttgactttttgcacttctgtatatagtcaaaagagagaaacctgtataatagtaagatc
ttattttgaataaaaacgtctataattacaaggagttttgttaaggctaatacaatgacagactgagcaa
aattgcttgcaaaagtggcacagagttagcactccatacccttcaaacatgttgctttgctttcttgtg
gacagcttgtagtttgccaggatttttcagctggaaagatacgccatcctttcaaaccctcatgactga
caaaaactccatggggccaaatctgcctgaagatcattaccaaaaatagcaggtacttctaccattaagg
tgaaatcatggatcagatattccttacatttttcaaaactactgcatgtttaaaacttcaacaaaaaaag
agagaaagaactatactaagaacatatattattcagatcagtttctgccaatttcagtggtttattgttc
acaaaaaaatcttcaaaacaagtattgactttcacaaaatttaaatcataaacaggcaaaccaaacagca
cactgtagctatagttgttatgtgattgtttttaattgctgtaggatcctgttctttcagcaggtgaaa
aataaaacgcagttcaaatttcatggtttaattttcaactcagaagcactcaaaaatgcaaaatgtgat
aatgggcacttgtttaaaagaattagtgtatccagccttcactccagctggttaaaaatgttgcacttat
cagcaaccctaccactttcatctgctgaaaggacaaatgtgcttggttttactattatgtaatcacaact
tactttctgcttgtagttgcttaaaattatgtattttgtcttgggctgcaatttgttttatgcttatttt
attattactgcagtagttgactttgctgtatggaaaaataaagtgaaattgccctaataaaacttctctt
tcttaagtaaaaaaaaaaaaaaaaaaaaa

> MBNL1 96 mut
ccacaagtatgttacccagatgtagaattttcatcactaaacaatcatgctaaagaggaaaggacagtgt
gcttggttagagtaaaggacgaggtcattagccatattgtatatatcgtcaagcaacacacacaaaagtt
cctcagccacaagacatccacatattgcatgttaaccagaagaaaagacaacattttccggaaatccact
gcacactgttgcctatacactttgtacatttaattgatatttgtgctgaggtgatattcctgtctaaaag
aacaacattgtctttcttttctagcacagagttatgcattcaaagatgcatacctagttagtttcctata
tattcatgccatcttgaaaagacagactatggtgtaaccatgattctattatgtattggtacgtctgtag
accaagatataatttttaaaaataagtttatttctttcaaggtttacaaataacaaaggtgcaccttgt
atttaaaattgccattatagatgagagcgtgcatgcacagtcatttttgtttaagagtaatatttttaat
gtaatagattgtaagacgtggtgagggagggatctgacagagatgaatgtgccaagcaaaaccacaactg

tgtatattttaaagcacatcatggctttaagtaccatgttgttaaggattctcatgaagtgccatagact
gtacatcaaattagagtattatttcttcagtgttattgttttcagagccacattttgttgcatatttgct
agtactaatcagtcaaagggcaccattctttttttttttttttgaaaccaaagctgtctcagaaatggcc
aatttaactttacagtaacaatagacagcacaacacaaactctctcaatacagataaactcacacatact
ggagatatatatataatagatatatataaaattattttaatgcattgtagtgtaatatttatgcatacta
tactgtataacatgttattcaaaagggattgccatttctgagacacagtaacaaaaaaatgaggaaatta
ttttgcttctatttatagcctctgtcaaaagtcaaaagactataaatgctttgcaaaaatggtttcacgt
ttgcttaaatgcttcatcacagtcacattcaaaatagtgactctaaacaaagaagaaagcagcactgtca
tcagatgcatgataaaccaaaatatgaaaatgggaaatgtttaattaacctagtaattgggtgggttaag
tacatgggtgaattttatatgtgattttgttttgttttgttttgttcagattaactgcttatagcctta
gaaagccttttacaaaattaaaaaaaaaatagatgtgcattcagtttttaagaatggaatcatccaaagg
aattcctttttttgaggtttggatgttgcagctagtaaaggatatttttgctctgttcagcagttctaaa
aattgctgaagtaggggccaggtcactggtagttatagtatggaatgggagaagtgaaagttcagttata
gaactttccatacttccaagtttactgcaagttttatgcttgagagagatgctttctaatataagactg
atgtgttgatttactgattgtactgtacatctattaaagccttagattattacattacgggttggaacc
cataccaatgtaatttcaatcgtgttaagaaagtaatggtgacttcacatgttattgtagttagttacat
tatagaatattacttatttttcttgttaaaatgtagtttttcatttcctacatttattagattttcattt
tctattaacaattgaataccatttcagtttatagacttgttttattagattttaccaatgaatttttcaa
aatacaaaaaaaagtagtttttccttcataacatactcagttttgaattacatgtagtgtcacatgaata
ttcgtattgttaactaaatgatttatattttactgatttaatattacagtgtaagaatgtcagtcattgt
tagttcttgtctagttttcattaaaagaacaaagatcttttatatggatatcttataaatatataatcat
tgctaagtaagaagttaagttgttgctatcgcaacaatcctggcagacaattgagtaatattttgatgat
ttattttgtttgtaattagttattataagaagatctagatcctagatattagaataaaatttattttcta
ctgtatccatttcaaatgttaaaatattgtttaatattttgaaatccctgagtatcaggccttgttata
aataagctgcataatcaataaatagaacaagggactttttgttgataatccaaatactcaaagtttacgt
aatgaaaattatagcgtgtgtgcaaactcttgagggttgattatgctgcaatttagcatgttggaacgtc
tagggagaaggttgacttttgcacttctgtatatagtcaaaagagagaaacctgtataatagtaagatc
ttattttgaataaaaacgtctataattacaaggagttttgttaaggctaatacaatgacagactgagcaa
aattgcttgcaaaagtggcacagagttagcactccataccccttcaaacatgttgctttgctttcttgtg
gacagcttgtagtttgccaggatttttcagctggaaagatacgccatcctttcaaaccctcatgactga
caaaaactccatggggccaaatctgcctgaagatcattaccaaaaatagcaggtacttctaccattaagg
tgaaatcatggatcagatattccttacattttcaaaactactgcatgtttaaaacttcaacaaaaaaag
agagaaagaactatactaagaacatatattattcagatcagtttctgccaatttcagtggtttattgttc
acaaaaaaatcttcaaaacaagtattgactttcacaaaatttaaatcataaacaggcaaaccaaacagca
cactgtagctatagttgttatgtgattgtttttaattgctgtaggatcctgttctttcagcaggtgaaa
aataaaacgcagttcaaatttcatggttttaattttcaactcagaagcactcaaaaatgcaaaatgtgat
aatgggcacttgtttaaaagaattagtgtatccagccttcactccagctggttaaaaatgttgcacttat
cagcaaccctaccactttcatctgctgaaaggacaaatgtgcttggttttactattatgtaatcacaact
tactttctgcttgtagttgcttaaaattatgtattttgtcttgggctgcaatttgttttatgcttatttt
attattactgcagtagttgactttgctgtatggaaaaataaagtgaaattgccctaataaaacttctctt
tcttaagtaaaaaaaaaaaaaaaaaaaaa

> MBNL1 181c mut1
ccacaagtatgttacccagatgtagaattttcatcactaaacaatcatgctaaagaggaaaggacagtgt
gcttggttagagtaaaggacgaggtcattagccatattgtatatatcgtcaagcaacacacacaaaagtt
cctcagccacaagacatccacatattgcatgttaaccagaagaaaagacaacattttccggaaatccact
gcacactgttgcctatacactttgtacatttaattgatatttgtgctgaggtgatattcctgtctaaaag
aacaacattgtctttcttttctagcacagagttatgcattcaaagatgcatacctagttagtttcctata
tattcatgccatcttgaaaagacagactatggtgtaaccatgattctattatgtattggtacgtctgtag
accaagatataatttttttaaaaataagtttatttctttcaaggtttacaaataacaaaggtgcaccttgt
atttaaaattgccattatagatgagagcgtgcatgcacagtcatttttgtttaagagtaatatttttaat
gtaatagattgtaagacgtggtgagggagggatctgacagagatgaatgtgccaagcaaaaccacaactg
tgtatattttaaagcacatcatggctttaagtaccatgttgttaaggattctcatgaagtgccatagact
gtacatcaaattagagtattatttcttcagtgttattgttttcagagccacattttgttgcatatttgct
agtactaatcagtcaaagggcaccattctttttttttttttttgaaaccaaagctgtctcagaaatggcc
aatttaactttacagtaacaatagacagcacaacacaaactctctcaatacagataaactcacacatact
ggagatatatatataatagatatatataaaattattttaatgcattgtagtgtaatatttatgcatacta
tactgtataacatgttattcaaaagggattgccatttctgagacacagtaacaaaaaaatgaggaaatta
ttttgcttctatttatagcctctgtcaaaagtcaaaagactataaatgctttgcaaaaatggtttcacgt
ttgcttaaatgcttcatcacagtcacattcaaaatagtgactctaaacaaagaagaaagcagcactgtca
tcagatgcatgataaaccaaaatatgaaaatgggaaatgtttaattaacctagtaattgggtgggttaag

tacatgggtgaattttatatgtgatttttgttttgttttgttttgttcagattaactgcttatagcctta
gaaagccttttacaaaattaaaaaaaaaatagatgtgcattcagtttttaagaatggaatcatccaaagg
aattcctttttttgaggtttggatgttgcagctagtaaaggatatttttgctctgttcagcagttctaaa
aattgctgaagtaggggccaggtcactggtagttatagtatggaatgggagaagtgaaagttcagttata
gaactttccatacttccaagtttactgcaagtttttatgcttgagagagatgctttctaatataagactg
atgtgttgattttactgattgtactgtacatctattaaagccttagattattacattacgggttggaacc
cataccaatgtaatttcaatcgtgttaagaaagtaatggtgacttcacatgttattgtagttagttacat
tatagaatattacttatttttcttgttaaaatgtagtttttcatttcctacatttattagattttcattt
tctattaacaattgaataccatttcagtttatagacttgttttattagattttaccaatgaatttttcaa
aatacaaaaaaaagtagtttttccttcataacatactcagttttgaattacatgtagtgtcacatgaata
ttcgtattgttaactaaatgatttatattttactgatttaatattacagtgtaagaatgtcagtcattgt
tagttcttgtctagttttcattaaaagaacaaagatcttttatatggatatcttataaatatataatcat
tgctaagtaagaagttaagttgttgctatcgcaacaatcctggcagacaattgagtaatattttgatgat
ttattttgtttgtaattagttattataagaagatctagatcctagatattagaataaaatttattttcta
ctgtatccatttcaaatgttaaaatattgtttaatatttttgaaatccctgagtatcaggccttgttata
aataagctgcataatcaataaatagaacaagggactttttgttgataatccaaatactcaaagtttacgt
aatgaaaattatagcgtgtgtgcaaactcttgagggttgattatgctgcaatttagcatgttggaacgtc
tagggagaaggttgacttttttgcacttctgtatatagtcaaaagagagaaacctgtataatagtaagatc
ttattttgaataaaaacgtctataattacaaggagttttgttaaggctaatacaatgacagactgagcaa
aattgcttgcaaaagtggcacagagttagcactccataccccttcaaacatgttgctttgctttcttgtg
gacagcttgtagtttgccaggatttttcagctggaaagatacgccatcctttcaaaccctcatgactga
caaaaactccatggggccaaatctgcctgaagatcattaccaaaaatagcaggtacttctaccattaagg
tgaaatcatggatcagatattccttacattttcaaaactactgcatgtttaaaacttcaacaaaaaaag
agagaaagaactatactaagaacatatattattcagatcagtttctgccaatttcagtggtttattgttc
acaaaaaaatcttcaaaacaagtattgactttcacaaaatttaaatcataaacaggcaaaccaaacagca
cactgtagctatagttgttatgtgattgtttttaattgctgtaggatcctgttctttcagcaggtgaaa
aataaaacgcagttcaaatttcatggttttaattttcaactcagaagcactcaaaaatgcaaaatgtgat
aatgggcacttgtttaaaagaattagtgtatccagccttcactccagctggttaaaaatgttgcacttat
cagcaaccctaccactttcatctgctgaaaggacaaatgtgcttggttttactattatgtaatcacaact
tactttctgcttgtagttgcttaaaattatgtattttgtcttgggctgcaatttgttttatgcttatttt
attattactgcagtagttgactttgctgtatggaaaaataaagtgaaattgccctaataaaacttctctt
tcttaagtaaaaaaaaaaaaaaaaaaaa

> MBNL1 181c mut2
ccacaagtatgttacccagatgtagaattttcatcactaaacaatcatgctaaagaggaaaggacagtgt
gcttggttagagtaaaggacgaggtcattagccatattgtatatatcgtcaagcaacacacacaaaagtt
cctcagccacaagacatccacatattgcatgttaaccagaagaaaagacaacattttccggaaatccact
gcacactgttgcctatacactttgtacatttaattgatatttgtgctgaggtgatattcctgtctaaaag
aacaacattgtctttcttttctagcacagagttatgcattcaaagatgcatacctagttagtttcctata
tattcatgccatcttgaaaagacagactatggtgtaaccatgattctattatgtattggtacgtctgtag
accaagatataatttttaaaaataagtttatttctttcaaggtttacaaataacaaaggtgcaccttgt
atttaaaattgccattatagatgagagcgtgcatgcacagtcatttttgtttaagagtaatatttttaat
gtaatagattgtaagacgtggtgagggagggatctgacagagatgaatgtgccaagcaaaaccacaactg
tgtatattttaaagcacatcatggctttaagtaccatgttgttaaggattctcatgaagtgccatagact
gtacatcaaattagagtattatttcttcagtgttattgttttcagagccacatttgttgcatatttgct
agtactaatcagtcaaagggcaccattcttttttttttttttgaaaccaaagctgtctcagaaatggcc
aatttaactttacagtaacaatagacagcacaacacaaactctctcaatacagataaactcacacatact
ggagatatatatataatagatatatataaaattattttaatgcattgtagtgtaatatttatgcatacta
tactgtataacatgttattcaaaagggattgccatttctgagacacagtaacaaaaaaatgaggaaatta
ttttgcttctatttatagcctctgtcaaaagtcaaaagactataaatgctttgcaaaaatggtttcacgt
ttgcttaaatgcttcatcacagtcacattcaaaatagtgactctaaacaaagaagaaagcagcactgtca
tcagatgcatgataaaccaaaatatgaaaatgggaaatgtttaattaacctagtaattgggtgggttaag
tacatgggtgaattttatatgtgatttttgttttgttttgttttgttcagattaactgcttatagcctta
gaaagccttttacaaaattaaaaaaaaaatagatgtgcattcagtttttaagaatggaatcatccaaagg
aattcctttttttgaggtttggatgttgcagctagtaaaggatatttttgctctgttcagcagttctaaa
aattgctgaagtaggggccaggtcactggtagttatagtatggaatgggagaagtgaaagttcagttata
gaactttccatacttccaagtttactgcaagtttttatgcttgagagagatgctttctaatataagactg
atgtgttgattttactgattgtactgtacatctattaaagccttagattattacattacgggttggaacc
cataccaatgtaatttcaatcgtgttaagaaagtaatggtgacttcacatgttattgtagttagttacat
tatagaatattacttatttttcttgttaaaatgtagtttttcatttcctacatttattagattttcattt
tctattaacaattgaataccatttcagtttatagacttgttttattagattttaccaatgaatttttcaa

aatacaaaaaaaagtagtttttccttcataacatactcagttttgaattacatgtagtgtcacatgaata
ttcgtattgttaactaaatgatttatattttactgatttaatattacagtgtaagaatgtcagtcattgt
tagttcttgtctagttttcattaaaagaacaaagatcttttatatggatatcttataaatatataatcat
tgctaagtaagaagttaagttgttgctatcgcaacaatcctggcagacaattgagtaatattttgatgat
ttattttgtttgtaattagttattataagaagatctagatcctagatattagaataaaatttattttcta
ctgtatccatttcaaatgttaaaatattgtttaatatttttgaaatccctgagtatcaggccttgttata
aataagctgcataatcaataaatagaacaagggactttttgttgataatccaaatactcaaagtttacgt
aatgaaaattatagcgtgtgtgcaaactcttgagggttgattatgctgcaatttagcatgttggaacgtc
tagggagaaggttgactttttgcacttctgtatatagtcaaaagagagaaacctgtataatagtaagatc
ttattttgaataaaaacgtctataattacaaggagttttgttaaggctaatacaatgacagactgagcaa
aattgcttgcaaaagtggcacagagttagcactccataccccttcaaacatgttgctttgctttcttgtg
gacagcttgtagtttgccaggatttttcagctggaaagatacgccatcctttcaaaccctcatgactga
caaaaactccatggggccaaatctgcctgaagatcattaccaaaaatagcaggtacttctaccattaagg
tgaaatcatggatcagatattccttacattttcaaaactactgcatgtttaaaacttcaacaaaaaaag
agagaaagaactatactaagaacatatattattcagatcagtttctgccaatttcagtggtttattgttc
acaaaaaaatcttcaaaacaagtattgactttcacaaaatttaaatcataaacaggcaaaccaaacagca
cactgtagctatagttgttatgtgattgtttttaattgctgtaggatcctgttctttcagcaggtgaaa
aataaaacgcagttcaaatttcatggttttaattttcaactcagaagcactcaaaaatgcaaaatgtgat
aatgggcacttgtttaaaagaattagtgtatccagccttcactccagctggttaaaaatgttgcacttat
cagcaaccctaccactttcatctgctgaaaggacaaatgtgcttggttttactattatgtaatcacaact
tactttctgcttgtagttgcttaaaattatgtattttgtcttgggctgcaatttgttttatgcttatttt
attattactgcagtagttgactttgctgtatggaaaaataaagtgaaattgccctaataaaacttctctt
tcttaagtaaaaaaaaaaaaaaaaaaaa

> MBNL1 181c mut3
ccacaagtatgttacccagatgtagaattttcatcactaaacaatcatgctaaagaggaaaggacagtgt
gcttggttagagtaaaggacgaggtcattagccatattgtatatatcgtcaagcaacacacacaaaagtt
cctcagccacaagacatccacatattgcatgttaaccagaagaaaagacaacattttccggaaatccact
gcacactgttgcctatacactttgtacatttaattgatatttgtgctgaggtgatattcctgtctaaaag
aacaacattgtctttcttttctagcacagagttatgcattcaaagatgcatacctagttagtttcctata
tattcatgccatcttgaaaagacagactatggtgtaaccatgattctattatgtattggtacgtctgtag
accaagatataatttttaaaaataagtttatttctttcaaggtttacaaataacaaaggtgcaccttgt
atttaaaattgccattatagatgagagcgtgcatgcacagtcatttttgtttaagagtaatattttaat
gtaatagattgtaagacgtggtgagggagggatctgacagagatgaatgtgccaagcaaaccacaactg
tgtatattttaaagcacatcatggctttaagtaccatgttgttaaggattctcatgaagtgccatagact
gtacatcaaattagagtattatttcttcagtgttattgttttcagagccacatttgttgcatatttgct
agtactaatcagtcaaagggcaccattcttttttttttttttgaaaccaaagctgtctcagaaatggcc
aatttaactttacagtaacaatagacagcacaacacaaactctctcaatacagataaactcacacatact
ggagatatatatataatagatatatataaaattattttaatgcattgtagtgtaatatttatgcatacta
tactgtataacatgttattcaaaagggattgccatttctgagacacagtaacaaaaaaatgaggaaatta
ttttgcttctatttatagcctctgtcaaaagtcaaaagactataaatgctttgcaaaaatggtttcacgt
ttgcttaaatgcttcatcacagtcacattcaaaatagtgactctaaacaaagaagaaagcagcactgtca
tcagatgcatgataaaccaaaatatgaaaatgggaaatgtttaattaacctagtaattgggtgggttaag
tacatgggtgaattttatatgtgattttgttttgttttgttttgttcagattaactgcttatagcctta
gaaagccttttacaaaattaaaaaaaaaatagatgtgcattcagtttttaagaatggaatcatccaaagg
aattcctttttttgaggtttggatgttgcagctagtaaaggatatttttgctctgttcagcagttctaaa
aattgctgaagtaggggccaggtcactggtagttatagtatggaatgggagaagtgaaagttcagttata
gaactttccatacttccaagtttactgcaagtttttatgcttgagagagatgctttctaatataagactg
atgtgttgattttactgattgtactgtacatctattaaagccttagattattacattacgggttggaacc
cataccaatgtaatttcaatcgtgttaagaaagtaatggtgacttcacatgttattgtagttagttacat
tatagaatattacttatttttcttgttaaaatgtagtttttcatttcctacatttattagattttcattt
tctattaacaattgaataccatttcagtttatagacttgttttattagattttaccaatgaattttttcaa
aatacaaaaaaaaagtagtttttccttcataacatactcagttttgaattacatgtagtgtcacatgaata
ttcgtattgttaactaaatgatttatattttactgatttaatattacagtgtaagaatgtcagtcattgt
tagttcttgtctagttttcattaaaagaacaaagatcttttatatggatatcttataaatatataatcat
tgctaagtaagaagttaagttgttgctatcgcaacaatcctggcagacaattgagtaatattttgatgat
ttattttgtttgtaattagttattataagaagatctagatcctagatattagaataaaatttattttcta
ctgtatccatttcaaatgttaaaatattgtttaatatttttgaaatccctgagtatcaggccttgttata
aataagctgcataatcaataaatagaacaagggactttttgttgataatccaaatactcaaagtttacgt
aatgaaaattatagcgtgtgtgcaaactcttgagggttgattatgctgcaatttagcatgttggaacgtc
tagggagaaggttgactttttgcacttctgtatatagtcaaaagagagaaacctgtataatagtaagatc

ttattttgaataaaaacgtctataattacaaggagttttgttaaggctaatacaatgacagactgagcaa
aattgcttgcaaaagtggcacagagttagcactccataccccttcaaacatgttgctttgctttcttgtg
gacagcttgtagtttgccaggatttttcagctggaaagatacgccatcctttcaaaccctcatgactga
caaaaactccatggggccaaatctgcctgaagatcattaccaaaaatagcaggtacttctaccattaagg
tgaaatcatggatcagatattccttacattttcaaaactactgcatgtttaaaacttcaacaaaaaaag
agagaaagaactatactaagaacatatattattcagatcagtttctgccaatttcagtggtttattgttc
acaaaaaaatcttcaaaacaagtattgactttcacaaaatttaaatcataaacaggcaaaccaaacagca
cactgtagctatagttgttatgtgattgtttttaattgctgtaggatcctgttctttcagcaggtgaaa
aataaaacgcagttcaaatttcatggttttaattttcaactcagaagcactcaaaaatgcaaaatgtgat
aatgggcacttgtttaaaagaattagtgtatccagccttcactccagctggttaaaaatgttgcacttat
cagcaaccctaccactttcatctgctgaaaggacaaatgtgcttggttttactattatgtaatcacaact
tactttctgcttgtagttgcttaaaattatgtattttgtcttgggctgcaatttgttttatgcttatttt
attattactgcagtagttgactttgctgtatggaaaaataaagtgaaattgccctaataaaacttctctt
tcttaagtaaaaaaaaaaaaaaaaaaaa

MUTAGENESIS:

> MBNL1 23b PM
ccacaagtatgttacccagatgtagaattttcatcactaaacaatcatgctaaagaggaaaggacagtgt
gcttggttagagtaaaggacgaggtcattagccatattgtatatatcgtcaagcaacacacacaaaagtt
cctcagccacaagacatccacatattgcatgttaaccagaagaaaagacaacattttccggaaatccact
gcacactgttgcctatacactttgtacatttaattgatatttgtgctgaggtgatattcctgtctaaaag
aacaacattgtctttcttttctagcacagagttatgcattcaaagatgcatacctagttagtttcctata
tattcatgccatcttgaaaagacagactatggtgtaaccatgattctattatgtattggtacgtctgtag
accaagatataatttttaaaaataagtttatttctttcaaggtttacaaataacaaaggtgcaccttgt
atttaaaattgccattatagatgagagcgtgcatgcacagtcatttttgtttaagagtaatatttttaat
gtaatagattgtaagacgtggtgagggagggatctgacagagatgaatgtgccaagcaaaaccacaactg
tgtatattttaaagcacatcatggctttaagtaccatgttgttaaggattctcatgaagtgccatagact
gtacatcaaattagagtattatttcttcagtgttattgttttcagagccacattttgttgcatatttgct
agtactaatcagtcaaagggcaccattctttttttttttttttgaaaccaaagctgtctcagaaatggcc
aatttaactttacagtaacaatagacagcacaacacaaactctctcaatacagataaactcacacatact
ggagatatatatataatagatatatataaaattattttaatgcattgtagtgtaatatttatgcatacta
tactgtataacatgttattcaaaagggattgccatttctgagacacagtaacaaaaaaatgaggaaatta
ttttgcttctatttatagcctctgtcaaaagtcaaaagactataaatgctttgcaaaaatggtttcacgt
ttgcttaaatgcttcatcacagtcacattcaaaatagtgactctaaacaaagaagaaagcagcactgtca
tcagatgcatgataaaccaaaatatgaaaatgggaaatgtttaattaacctagtaattgggtgggttaag
tacatgggtgaattttatatgtgattttttgttttgttttgttttgttcagattaactgcttatagcctta
gaaagccttttacaaaattaaaaaaaaaatagatgtgcattcagtttttaagaatggaatcatccaaagg
aattcctttttttgaggtttggatgttgcagctagtaaaggatatttttgctctgttcagcagttctaaa
aattgctgaagtaggggccaggtcactggtagttatagtatggaatgggagaagtgaaagttcagttata
gaactttccatacttccaagtttactgcaagttttatgcttgagagagatgctttctaatataagactg
atgtgttgattttactgattgtactgtacatctattaaagccttagattattacattacgggttggaacc
cataccaatgtaatttcaatcgtgttaagaaagtaatggtgacttcacatgttattgtagttagttacat
tatagaatattacttatttttcttgttaaaatgtagtttttcatttcctacatttattagattttcattt
tctattaacaattgaataccatttcagtttatagacttgttttattagattttaccaatgaattttcaa
aatacaaaaaaaagtagtttttccttcataacatactcagttttgaattacatgtagtgtcacatgaata
ttcgtattgttaactaaatgatttatattttactgatttaatattacagtgtaagaatgtcagtcattgt
tagttcttgtctagttttcattaaaagaacaaagatcttttatatggatatcttataaatatataatcat
tgctaagtaagaagttaagttgttgctatcgcaacaatcctggcagacaattgagtaatattttgatgat
ttattttgtttgtaattagttattataagaagatctagatcctagatattagaataaaatttattttcta
ctgtatccatttcaaatgttaaaatattgtttaatatttttgaaatccctgagtatcaggccttgttata
aataagctgcataatcaataaatagaacaagggactttttgttgataatccaaatactcaaagtttacgt
aatgaaaattatagcgtgtgtgcaaactcttgagggttgattatgctgcaatttagcatgttggaacgtc
tagggagaaggttgacttttttgcacttctgtatatagtcaaaagagagaaacctgtataatagtaagatc
ttattttgaataaaaacgtctataattacaaggagttttgttaaggctaatacaatgacagactgagcaa
aattgcttgcaaaagtggcacagagttagcactccataccccttcaaacatgttgctttgctttcttgtg
gacagcttgtagtttgccaggatttttcagctggaaagatacgccatcctttcaaaccctcatgactga
caaaaactccatggggccaaatctgcctgaagatcattaccaaaaatagcaggtacttctaccattaagg
tgaaatcatggatcagatattccttacattttcaaaactactgcatgtttaaaacttcaacaaaaaaag
agagaaagaactatactaagaacatatattattcagatcagtttctgccaatttcagtggtttattgttc
acaaaaaaatcttcaaaacaagtattgactttcacaaaatttaaatcataaacaggcaaaccaaacagca

cactgtagctatagttgttatgtgattgttttttaattgctgtaggatcctgttctttcagcaggtgaaa
aataaaacgcagttcaaatttcatggttttaattttcaactcagaagcaGGTaaTCCCTGGCaatgtgat
aatgggcacttgtttaaaagaattagtgtatccagccttcactccagctggttaaaaatgttgcacttat
cagcaaccctaccactttcatctgctgaaaggacaaatgtgcttggttttactattatgtaatcacaact
tactttctgcttgtagttgcttaaaattatgtattttgtcttgggctgcaatttgttttatgcttatttt
attattactgcagtagttgactttgctgtatggaaaaataaagtgaaattgccctaataaaacttctctt
tcttaagtaaaaaaaaaaaaaaaaaaaa

> MBNL1 96 PM
ccacaagtatgttacccagatgtagaattttcatcactaaacaatcatgctaaagaggaaaggacagtgt
gcttggttagagtaaaggacgaggtcattagccatattgtatatatcgtcaagcaacacacacaaaagtt
cctcagccacaagacatccacatattgcatgttaaccagaagaaaagacaacattttccggaaatccact
gcacactgttgcctatacactttgtacatttaattgatatttgtgctgaggtgatattcctgtctaaaag
aacaacattgtctttcttttctagcacagagttatgcattcaaagatgcatacctagttagtttcctata
tattcatgccatcttgaaaagacagactatggtgtaaccatgattctattatgtattggtacgtctgtag
accaagatataattttttaaaaataagtttatttctttcaaggtttacaaataacaaaggtgcaccttgt
atttaaaattgccattatagatgagagcgtgcatgcacagtcatttttgtttaagagtaatatttttaat
gtaatagattgtaagacgtggtgagggagggatctAGcaAaAatgTGCTAgtgccaaAcaaaaccacaac
tgtgtatattttaaagcacatcatggctttaagtaccatgttgttaaggattctcatgaagtgccataga
ctgtacatcaaattagagtattatttcttcagtgttattgttttcagagccacattttgttgcatatttg
ctagtactaatcagtcaaagggcaccattctttttttttttttttgaaaccaaagctgtctcagaaatgg
ccaatttaactttacagtaacaatagacagcacaacacaaactctctcaatacagataaactcacacata
ctggagatatatatataatagatatatataaaattattttaatgcattgtagtgtaatatttatgcatac
tatactgtataacatgttattcaaaagggattgccatttctgagacacagtaacaaaaaaatgaggaaat
tattttgcttctatttatagcctctgtcaaaagtcaaaagactataaatgctttgcaaaaatggtttcac
gtttgcttaaatgcttcatcacagtcacattcaaaatagtgactctaaacaaagaagaaagcagcactgt
catcagatgcatgataaaccaaaatatgaaaatgggaaatgtttaattaacctagtaattgggtgggtta
agtacatgggtgaattttatatgtgatttttgttttgttttgttttgttcagattaactgcttatagcct
tagaaagccttttacaaaattaaaaaaaaaatagatgtgcattcagtttttaagaatggaatcatccaaa
ggaattcctttttttgaggtttggatgttgcagctagtaaaggatatttttgctctgttcagcagttcta
aaaattgctgaagtaggggccaggtcactggtagttatagtatggaatgggagaagtgaaagttcagtta
tagaactttccatacttccaagtttactgcaagtttttatgcttgagagagatgctttctaatataagac
tgatgtgttgattttactgattgtactgtacatctattaaagccttagattattacattacgggttggaa
cccataccaatgtaatttcaatcgtgttaagaaagtaatggtgacttcacatgttattgtagttagttac
attatagaatattacttatttttcttgttaaaatgtagtttttcatttcctacatttattagattttcat
tttctattaacaattgaataccatttcagtttatagacttgttttattagattttaccaatgaatttttc
aaaatacaaaaaaaagtagtttttccttcataacatactcagttttgaattacatgtagtgtcacatgaa
tattcgtattgttaactaaatgatttatattttactgatttaatattacagtgtaagaatgtcagtcatt
gttagttcttgtctagttttcattaaaagaacaaagatcttttatatggatatcttataaatatataatc
attgctaagtaagaagttaagttgttgctatcgcaacaatcctggcagacaattgagtaatattttgatg
atttattttgtttgtaattagttattataagaagatctagatcctagatattagaataaaatttattttc
tactgtatccatttcaaatgttaaaatattgtttaatatttttgaaatccctgagtatcaggccttgtta
taaataagctgcataatcaataaatagaacaagggactttttgttgataatccaaatactcaaagtttac
gtaatgaaaattatagcgtgtgtgcaaactcttgagggttgattatgctgcaatttagcatgttggaacg
tctagggagaaggttgactttttgcacttctgtatatagtcaaaagagagaaacctgtataatagtaaga
tcttattttgaataaaaacgtctataattacaaggagttttgttaaggctaatacaatgacagactgagc
aaaattgcttgcaaaagtggcacagagttagcactccataccccttcaaacatgttgctttgctttcttg
tggacagcttgtagtttgccaggattttttcagctggaaagatacgccatcctttcaaaccctcatgact
gacaaaaactccatggggccaaatctgcctgaagatcattaccaaaaatagcaggtacttctaccattaa
ggtgaaatcatggatcagatattccttacatttttcaaaactactgcatgtttaaaacttcaacaaaaaa
agagagaaagaactatactaagaacatatattattcagatcagtttctgccaatttcagtggtttattgt
tcacaaaaaaatcttcaaaacaagtattgactttcacaaaatttaaatcataaacaggcaaaccaaacag
cacactgtagctatagttgttatgtgattgttttttaattgctgtaggatcctgttctttcagcaggtga
aaaataaaacgcagttcaaatttcatggttttaattttcaactcagaagcactcaaaaatgcaaaatgtg
ataatgggcacttgtttaaaagaattagtgtatccagccttcactccagctggttaaaaatgttgcactt
atcagcaaccctaccactttcatctgctgaaaggacaaatgtgcttggttttactattatgtaatcacaa
cttactttctgcttgtagttgcttaaaattatgtattttgtcttgggctgcaatttgttttatgcttatt
ttattattactgcagtagttgactttgctgtatggaaaaataaagtgaaattgccctaataaaacttctc
tttcttaagtaaaaaaaaaaaaaaaaaaaa

> MBNL1 181c PM

ccacaagtatgttacccagatgtagaattttcatcactaaacaatcatgctaaagaggaaaggacagtgt
gcttggttagagtaaaggacgaggtcattagccatattgtatatatcgtcaagcaacacacacaaaagtt
cctcagccacaagacatccacatattgcatgttaaccagaagaaaagacaacattttccggaaatccact
gcacactgttgcctatacactttgtacatttaattgatatttgtgctgaggtgatattcctgtctaaaag
aacaacattgtctttcttttctagcacagagttatgcattcaaagatgcatacctagttagtttcctata
tattcatgccatcttgaaaagacagactatggtgtaaccatgattctattatgtattggtacgtctgtag
accaagatataattttttaaaaataagtttatttctttcaaggtttacaaataacaaaggtgcaccttgt
atttaaaattgccattatagatgagagcgtgcatgcacagtcatttttgtttaagagtaatattttttaat
gtaatagattgtaagacgtggtgagggaACTCACCGACAGGTTGAATGTTccaagcaaaaccacaactgt
gtatattttaaagcacatcatggctttaagtaccatgttgttaaggattctcatgaagtgccatagactg
tacatcaaattagagtattatttcttcagtgttattgttttcagagccacattttgttgcatatttgcta
gtactaatcagtcaaagggcaccattctttttttttttttttgaaaccaaagctgtctcagaaatggcca
atttaactttacagtaacaatagacagcacaacacaaactctctcaatacagataaactcacacatactg
gagatatatatataatagatatatataaaattattttaatgcattgtagtgtaatatttatgcatactat
actgtataacatgttattcaaaagggattgccatttctgagacacagtaacaaaaaaatgaggaaattat
tttgcttctatttatagcctctgtcaaaagtcaaaagactataaatgctttgcaaaaatggtttcacgtt
tgcttaaatgcttcatcacagtcacattcaaaatagtgactctaaacaaagaagaaagcagcactgtcat
cagatgcatgataaaccaaaatatgaaaatgggaaatgtttaattaacctagtaattgggtgggttaagt
acatgggtgaattttatatgtgatttttgttttgttttgttttgttcagattaactgcttatagccttag
aaagccttttacaaattaaaaaaaaaaatagatgtgcattcagtttttaagaatggaatcatccaaagga
attcACTCACCGACAGGTTGAATGTTgcagctagtaaaggatattttgctctgttcagcagttctaaaa
attgctgaagtaggggccaggtcactggtagttatagtatggaatgggagaagtgaaagttcagttatag
aactttccatacttccaagtttactgcaagtttttatgcttgagagagatgctttctaatataagactga
tgtgttgattttactgattgtactgtacatctattaaagccttagattattacattacgggttggaaccc
ataccaatgtaatttcaatcgtgttaagaaagtaatggtgacttcacatgttattgtagttagttacatt
atagaatattacttatttttcttgttaaaatgtagtttttcatttcctacatttattagattttcatttt
ctattaacaattgaataccatttcagtttatagacttgttttattagattttaccaatgaatttttcaaa
atacaaaaaaaagtagtttttccttcataacatactcagttttgaattacatgtagtgtcacatgaatat
tcgtattgttaactaaatgatttatattttactgatttACTCACCGACAGGTTGAATGTTagtcattgtt
agttcttgtctagttttcattaaaagaacaaagatcttttatatggatatcttataaatatataatcatt
gctaagtaagaagttaagttgttgctatcgcaacaatcctggcagacaattgagtaatattttgatgatt
tattttgtttgtaattagttattataagaagatctagatcctagatattagaataaaatttattttctac
tgtatccatttcaaatgttaaaatattgtttaatattttgaaatccctgagtatcaggccttgttataa
ataagctgcataatcaataaatagaacaagggacttttgttgataatccaaatactcaaagtttacgta
atgaaaattatagcgtgtgtgcaaactcttgagggttgattatgctgcaatttagcatgttggaacgtct
agggagaaggttgacttttgcacttctgtatatagtcaaaagagagaaacctgtataatagtaagatct
tattttgaataaaaacgtctataattacaaggagttttgttaaggctaatacaatgacagactgagcaaa
attgcttgcaaaagtggcacagagttagcactccataccccttcaaacatgttgctttgctttcttgtgg
acagcttgtagtttgccaggatttttcagctggaaagatacgccatcctttcaaaccctcatgactgac
aaaaactccatggggccaaatctgcctgaagatcattaccaaaaatagcaggtacttctaccattaaggt
gaaatcatggatcagatattccttacattttcaaaactactgcatgtttaaaacttcaacaaaaaaaga
gagaaagaactatactaagaacatatattattcagatcagtttctgccaatttcagtggtttattgttca
caaaaaaatcttcaaaacaagtattgactttcacaaaatttaaatcataaacaggcaaaccaaacagcac
actgtagctatagttgttatgtgattgtttttaattgctgtaggatcctgttctttcagcaggtgaaaa
ataaaacgcagttcaaatttcatggttttaattttcaactcagaagcactcaaaaatgcaaaatgtgata
atgggcacttgtttaaaagaattagtgtatccagccttcactccagctggttaaaaatgttgcacttatc
agcaaccctaccactttcatctgctgaaaggacaaatgtgcttggttttactattatgtaatcacaactt
actttctgcttgtagttgcttaaaattatgtattttgtcttgggctgcaatttgttttatgcttatttta
ttattactgcagtagttgactttgctgtatggaaaaataaagtgaaattgccctaataaaacttctcttt
cttaagtaaaaaaaaaaaaaaaaaaaaa

B) MBNL2

ORIGINAL SEQUENCE (in pEZX-MT05):

tcagcagaaacggaatggaatgccaagaatctgcattgagaataactaaacattgttactgtacatacta
tcctgtttcctcctcaatagaattgccacaaactgcatgctaaataaagatgtagttcttctggacagac
cacaactctaagaagctagtgctgctatctcatatatgagtattaaatatggtatgcttagtatattcca
acctaagatagttaactacctgagaccagctgtgatgtttaaagacataaaggataaagtttacttttaa
agggtttctaaacatagtttctgtcctaggaatattgtcttatctccataactatagctgatgcagaaag

tccagccagtttactcatttcgattcagaatatttcaaatttagcaataaacaattagcattagttaaaa
aagaaacatattccaagggcaggttcgattctagctctaattactgtcatgtcatttacccactggatca
aagggtatgtttcacttcttgacaatataaatgctgcagcaaagatgagaggtgaagtaaaaccgatacc
tgtcctgcaggtctaaaatttgaatggaaattcaagcacaagtactggggacacatcaaagtgtggtgtt
tggtttgcctggagatgccacgttgaatcatgtgattctagattaacattaaatagattgaaaaagaaac
tttgcacggtatgagcttcataccccaccaaacaaagtcttgaaggtattattttacaagtatattttta
aagttgttttataagagagactttgtagaagtgcctagattttgccagacttcatccagcttgacaagat
tgagaggcccatgccaacagtctaatctaagagattagtctttcaaactcaccatccagttgcctgttac
agaataactcttcttaactaaaaacctagtcaaacaaggaagctgtaggtgaggagatctgtataatatt
ctaatttaagtaagtttgagtttagtcactgcaaatttgactgtgactttaatctaaattactatgtaaa
caaaaagtagatagtttcacttttaaaaaatccattactgttttgcatttcaaaagttggattaaaggg
ttgtaactgactacagcatggaaaaaaatagttcttttaattctttcaccttaaagcatattttatgtct
caaaagtataaaaaactttaatacaagtacatacatattatatatacacatacatatatatactatatat
ggatgaaacatattttaatgttgtttactttttaaatacttggttgatcttcaaggtaatagcgataca
attaaatttgttcagaaagtttgttttaaagtttattttaagcactatcgtaccaaatatttcatattt
cacattttatatgttgcacatagcctatacagtacctacatagtttttaaattattgtttaaaaaacaaa
acagctgttataaatgaatattatgtgtaattgtttcaaacatccattttctttgtgaacatattagtga
ttgaagtattttgacttttgagattgaatgtaaaatattttaaatttgggatcatcgcctgttctgaaaa
ctagatgcaccaaccgtatcattatttgtttgaggaaaaaaagaaatctgcattttaattcatgttggtc
aaagtcgaattactatctatttatcttatatcgtagatctgataaccctatctaaaagaaagtcacacgc
taaatgtattcttacatagtgcttgtatcgttgcatttgttttaatttgtggaaaagtattgtatctaac
ttgtattactttggtagtttcatctttatgtattattgatatttgtaattttctcaactataacaatgta
gttacgctacaacttgcctaaaacattcaaacttgtttctttttctgtttttttctttgttaattcat
ttaaactcattgaaaacatagtatacattactaaaaggtaaattatgggaatcactgaaatattttgta
gattaattgttgtaacattgtctttctttttttctttgtttcatgattttgatttttaaaattattag
cacacaactattttcagccctttaataatggagcatcaaaaacatcacctgtaaccccaagcaaatatag
aagactgtatttttttactatgatatccattttccagaattgtgattacaatatgcaaagagtcataaata
tgccatttacaataaggaggaggcaaggcaaatgcatagatgtacaaatatatgtacaacagattttgct
ttttatttatttataatgtaattttatagaataattctgggatttgagaggatctaaaactattttctg
tataaatattatttgccaaaagtttgtttatattcagaagtctgactatgatgaataaatcttaaatgct
ttgtttaattaaaaaacaaaaatcaccaatatccaagacatgaagatatcagttcaacaaatactgtagt
taagagactaactctccacttgtatgggaactacatttcactcttggttttcaggatataacagcacttc
accgaaatattctttcagccataccactggtaacatttctactaaatctttctgtaacacttaaagaatt
ccctcattcattaccttacagtgtaaacaggagtctaatttgtatcaatactatgttttggttgtaatat
tcagttcactcacccaatgtacaaccaatgaaataaaagaagcatttaaaaggaaaaaaaaaaaaaaaaa
aaaaaaaaaaa

DELETIONS:

> MBNL1 218 mut1
tcagcagaaacggaatggaatgccaagaatctgcattgagaataactaaacattgttactgtacatacta
tcctgtttcctcctcaatagaattgccacaaactgcatgctaaataaagatgtagttcttctggacagac
cacaactctaagaagctagtgctgctatctcatatatgagtattaaatatggtatgcttagtatattcca
acctaagatagttaactacctgagaccagctgtgatgtttaaagacataaaggataaagtttacttttaa
agggtttctaaacatagtttctgtcctaggaatattgtcttatctccataactatagctgatgcagaaag
tccagccagtttactcatttcgattcagaatatttcaaatttagcaataaacaattagcattagttaaaa
aagaaacatattccaagggcaggttcgattctagctctaattactgtcatgtcatttacccactggatca
aagggtatgtttcacttcttgacaatataaatgctgcagcaaagatgagaggtgaagtaaaaccgatacc
tgtcctgcaggtctaaaatttgaatggaaattcaagcacaagtactggggacacatcaaagtgtggtgtt
tggtttgcctggagatgccacgttgaatcatgtgattctagattaacattaaatagattgaaaaagaaac
tttgcacggtatgagcttcataccccaccaaacaaagtcttgaaggtattattttacaagtatattttta
aagttgttttataagagagactttgtagaagtgcctagattttgccagacttcatccagcttgacaagat
tgagaggcccatgccaacagtctaatctaagagattagtctttcaaactcaccatccagttgcctgttac
agaataactcttcttaactaaaaacctagtcaaacaaggaagctgtaggtgaggagatctgtataatatt
ctaatttaagtaagtttgagtttagtcactgcaaatttgactgtgactttaatctaaattactatgtaaa
caaaaagtagatagtttcacttttaaaaaatccattactgttttgcatttcaaaagttggattaaaggg
ttgtaactgactacagcatggaaaaaaatagttcttttaattctttcaccttaaagcatattttatgtct
caaaagtataaaaaactttaatacaagtacatacatattatatatacacatacatatatatactatatat
ggatgaaacatattttaatgttgtttactttttaaatacttggttgatcttcaaggtaatagcgataca
attaaatttgttcagaaagtttgttttaaagtttattttaagcactatcgtaccaaatatttcatattt
cacattttatatgttgcacatagcctatacagtacctacatagtttttaaattattgtttaaaaaacaaa

acagctgttataaatgaatattatgtgtaattgtttcaaacatccattttctttgtgaacatattagtga
ttgaagtattttgacttttgagattgaatgtaaaatattttaaatttgggatcatcgcctgttctgaaaa
ctagatgcaccaaccgtatcattatttgtttgaggaaaaaaagaaatctgcattttaattcatgttggtc
aaagtcgaattactatctatttatcttatatcgtagatctgataaccctatctaaaagaaagtcacacgc
taaatgtattcttacatagtgcttgtatcgttgcatttgttttaatttgtggaaaagtattgtatctaac
ttgtattactttggtagtttcatctttatgtattattgatatttgtaattttctcaactataacaatgta
gttacgctacaacttgcctaaaacattcaaacttgttttctttttctgtttttttctttgttaattcat
ttaaactcattgaaaacatagtatacattactaaaaggtaaattatgggaatcactgaaatatttttgta
gattaattgttgtaacattgtctttctttttttcttttgtttcatgattttgatttttaaaattattag
cacacaactattttcagccctttaataatggagcatcaaaaacatcacctgtaaccccaagcaaatatag
aagactgtatttttttactatgatatccattttccagaattgtgattacaatatgcaaagagtcataaata
tgccatttacaataaggaggaggcaaggcaaatgcatagatgtacaaatatatgtacaacagattttgct
ttttatttatttataatgtaattttatagaataattctgggatttgagaggatctaaaactatttttctg
tataaatattatttgccaaaagtttgtttatattcagaagtctgactatgatgaataaatcttaaatgct
ttgtttaattaaaaaacaaaaatcaccaatatccaagacatgaagatatcagttcaacaaatactgtagt
taagagactaactctccacttgtatgggaactacatttcactcttggttttcaggatataacagcacttc
accgaaatattctttcagccataccactggtaacatttctactaaatctttctgtaacacttaaagaatt
ccctcattcattaccttacagtgtaaacaggagtctaatttgtatcaatactatgttttggttgtaatat
tcagttcactcacccaatgtacaaccaatgaaataaaagaagcatttaaaaggaaaaaaaaaaaaaaaaa
aaaaaaaaaaa

> MBNL1 218 mut2
tcagcagaaacggaatggaatgccaagaatctgcattgagaataactaaacattgttactgtacatacta
tcctgtttcctcctcaatagaattgccacaaactgcatgctaaataaagatgtagttcttctggacagac
cacaactctaagaagctagtgctgctatctcatatatgagtattaaatatggtatgcttagtatattcca
acctaagatagttaactacctgagaccagctgtgatgtttaaagacataaaggataaagtttacttttaa
agggtttctaaacatagtttctgtcctaggaatattgtcttatctccataactatagctgatgcagaaag
tccagccagtttactcatttcgattcagaatatttcaaatttagcaataaacaattagcattagttaaaa
aagaaacatattccaagggcaggttcgattctagctctaattactgtcatgtcatttacccactggatca
aagggtatgtttcacttcttgacaatataaatgctgcagcaaagatgagaggtgaagtaaaaccgatacc
tgtcctgcaggtctaaaatttgaatggaaattcaagcacaagtactggggacacatcaaagtgtggtgtt
tggtttgcctggagatgccacgttgaatcatgtgattctagattaacattaaatagattgaaaaagaaac
tttgcacggtatgagcttcataccccaccaaacaaagtcttgaaggtattattttacaagtatattttta
aagttgttttataagagagactttgtagaagtgcctagattttgccagacttcatccagcttgacaagat
tgagaggcccatgccaacagtctaatctaagagattagtctttcaaactcaccatccagttgcctgttac
agaataactcttcttaactaaaaacctagtcaaacaaggaagctgtaggtgaggagatctgtataatatt
ctaatttaagtaagtttgagtttagtcactgcaaatttgactgtgactttaatctaaattactatgtaaa
caaaaagtagatagtttcacttttttaaaaaatccattactgttttgcatttcaaaagttggattaaaggg
ttgtaactgactacagcatggaaaaaaatagttcttttaattctttcaccttaaagcatattttatgtct
caaaagtataaaaaactttaatacaagtacatacatattatatatacacatacatatatatactatatat
ggatgaaacatattttaatgttgtttactttttttaaatacttggttgatcttcaaggtaatagcgataca
attaaattttgttcagaaagtttgttttaaagtttattttaagcactatcgtaccaaatatttcatattt
cacattttatatgttgcacatagcctatacagtacctacatagtttttaaattattgtttaaaaaacaaa
acagctgttataaatgaatattatgtgtaattgtttcaaacatccattttctttgtgaacatattagtga
ttgaagtattttgacttttgagattgaatgtaaaatattttaaatttgggatcatcgcctgttctgaaaa
ctagatgcaccaaccgtatcattatttgtttgaggaaaaaaagaaatctgcattttaattcatgttggtc
aaagtcgaattactatctatttatcttatatcgtagatctgataaccctatctaaaagaaagtcacacgc
taaatgtattcttacatagtgcttgtatcgttgcatttgttttaatttgtggaaaagtattgtatctaac
ttgtattactttggtagtttcatctttatgtattattgatatttgtaattttctcaactataacaatgta
gttacgctacaacttgcctaaaacattcaaacttgttttctttttctgtttttttctttgttaattcat
ttaaactcattgaaaacatagtatacattactaaaaggtaaattatgggaatcactgaaatatttttgta
gattaattgttgtaacattgtctttctttttttcttttgtttcatgattttgatttttaaaattattag
cacacaactattttcagccctttaataatggagcatcaaaaacatcacctgtaaccccaagcaaatatag
aagactgtatttttttactatgatatccattttccagaattgtgattacaatatgcaaagagtcataaata
tgccatttacaataaggaggaggcaaggcaaatgcatagatgtacaaatatatgtacaacagattttgct
ttttatttatttataatgtaattttatagaataattctgggatttgagaggatctaaaactatttttctg
tataaatattatttgccaaaagtttgtttatattcagaagtctgactatgatgaataaatcttaaatgct
ttgtttaattaaaaaacaaaaatcaccaatatccaagacatgaagatatcagttcaacaaatactgtagt
taagagactaactctccacttgtatgggaactacatttcactcttggttttcaggatataacagcacttc
accgaaatattctttcagccataccactggtaacatttctactaaatctttctgtaacacttaaagaatt
ccctcattcattaccttacagtgtaaacaggagtctaatttgtatcaatactatgttttggttgtaatat

tcagttcactcacccaatgtacaaccaatgaaataaaagaagcatttaaaggaaaaaaaaaaaaaaaa
aaaaaaaaaaa

> MBNL1 218 mut3
tcagcagaaacggaatggaatgccaagaatctgcattgagaataactaaacattgttactgtacatacta
tcctgtttcctcctcaatagaattgccacaaactgcatgctaaataaagatgtagttcttctggacagac
cacaactctaagaagctagtgctgctatctcatatatgagtattaaatatggtatgcttagtatattcca
acctaagatagttaactacctgagaccagctgtgatgtttaaagacataaaggataaagtttactttaa
agggtttctaaacatagtttctgtcctaggaatattgtcttatctccataactatagctgatgcagaaag
tccagccagtttactcatttcgattcagaatatttcaaatttagcaataaacaattagcattagttaaaa
aagaaacatattccaagggcaggttcgattctagctctaattactgtcatgtcatttacccactggatca
aagggtatgtttcacttcttgacaatataaatgctgcagcaaagatgagaggtgaagtaaaaccgatacc
tgtcctgcaggtctaaaatttgaatggaaattcaagcacaagtactggggacacatcaaagtgtggtgtt
tggtttgcctggagatgccacgttgaatcatgtgattctagattaacattaaatagattgaaaaagaaac
tttgcacggtatgagcttcataccccaccaaacaaagtcttgaaggtattattttacaagtatattttta
aagttgttttataagagagactttgtagaagtgcctagattttgccagacttcatccagcttgacaagat
tgagaggcccatgccaacagtctaatctaagagattagtctttcaaactcaccatccagttgcctgttac
agaataactcttcttaactaaaaacctagtcaaacaaggaagctgtaggtgaggagatctgtataatatt
ctaatttaagtaagtttgagtttagtcactgcaaatttgactgtgactttaatctaaattactatgtaaa
caaaaagtagatagtttcactttttaaaaaatccattactgttttgcatttcaaaagttggattaaaggg
ttgtaactgactacagcatggaaaaaaatagttcttttaattctttcaccttaaagcatattttatgtct
caaaagtataaaaaactttaatacaagtacatacatattatatatacacatacatatatatactatatat
ggatgaaacatattttaatgttgtttactttttaaatacttggttgatcttcaaggtaatagcgataca
attaaattttgttcagaaagtttgttttaaagtttattttaagcactatcgtaccaaatatttcatattt
cacattttatatgttgcacatagcctatacagtacctacatagtttttaaattattgtttaaaaaacaaa
acagctgttataaatgaatattatgtgtaattgtttcaaacatccattttctttgtgaacatattagtga
ttgaagtattttgacttttgagattgaatgtaaaatattttaaatttgggatcatcgcctgttctgaaaa
ctagatgcaccaaccgtatcattatttgtttgaggaaaaaaagaaatctgcattttaattcatgttggtc
aaagtcgaattactatctatttatcttatatcgtagatctgataaccctatctaaaagaaagtcacacgc
taaatgtattcttacatagtgcttgtatcgttgcatttgttttaatttgtggaaaagtattgtatctaac
ttgtattactttggtagtttcatctttatgtattattgatatttgtaattttctcaactataacaatgta
gttacgctacaacttgcctaaaacattcaaacttgtttcttttttctgtttttttctttgttaattcat
ttaaactcattgaaaacatagtatacattactaaaaggtaaattatgggaatcactgaaatattttgta
gattaattgttgtaacattgtctttctttttttctttgtttcatgattttgattttaaaattattag
cacacaactattttcagccctttaataatggagcatcaaaaacatcacctgtaaccccaagcaaatatag
aagactgtatttttactatgatatccattttccagaattgtgattacaatatgcaaagagtcataaata
tgccatttacaataaggaggaggcaaggcaaatgcatagatgtacaaatatatgtacaacagattttgct
ttttatttatttataatgtaattttatagaataattctgggatttgagaggatctaaaactatttttctg
tataaatattatttgccaaaagtttgtttatattcagaagtctgactatgatgaataaatcttaaatgct
ttgtttaattaaaaaacaaaaatcaccaatatccaagacatgaagatatcagttcaacaaatactgtagt
taagagactaactctccacttgtatgggaactacatttcactcttggttttcaggatataacagcacttc
accgaaatattctttcagccataccactggtaacatttctactaaatctttctgtaacacttaaagaatt
ccctcattcattaccttacagtgtaaacaggagtctaatttgtatcaatactatgttttggttgtaatat
tcagttcactcacccaatgtacaaccaatgaaataaaagaagcatttaaaggaaaaaaaaaaaaaaaa
aaaaaaaaaaa

> MBNL1 23b mut
tcagcagaaacggaatggaatgccaagaatctgcattgagaataactaaacattgttactgtacatacta
tcctgtttcctcctcaatagaattgccacaaactgcatgctaaataaagatgtagttcttctggacagac
cacaactctaagaagctagtgctgctatctcatatatgagtattaaatatggtatgcttagtatattcca
acctaagatagttaactacctgagaccagctgtgatgtttaaagacataaaggataaagtttactttaa
agggtttctaaacatagtttctgtcctaggaatattgtcttatctccataactatagctgatgcagaaag
tccagccagtttactcatttcgattcagaatatttcaaatttagcaataaacaattagcattagttaaaa
aagaaacatattccaagggcaggttcgattctagctctaattactgtcatgtcatttacccactggatca
aagggtatgtttcacttcttgacaatataaatgctgcagcaaagatgagaggtgaagtaaaaccgatacc
tgtcctgcaggtctaaaatttgaatggaaattcaagcacaagtactggggacacatcaaagtgtggtgtt
tggtttgcctggagatgccacgttgaatcatgtgattctagattaacattaaatagattgaaaaagaaac
tttgcacggtatgagcttcataccccaccaaacaaagtcttgaaggtattattttacaagtatattttta
aagttgttttataagagagactttgtagaagtgcctagattttgccagacttcatccagcttgacaagat
tgagaggcccatgccaacagtctaatctaagagattagtctttcaaactcaccatccagttgcctgttac
agaataactcttcttaactaaaaacctagtcaaacaaggaagctgtaggtgaggagatctgtataatatt

ctaatttaagtaagtttgagtttagtcactgcaaatttgactgtgactttaatctaaattactatgtaaa
caaaaagtagatagtttcactttttaaaaaatccattactgttttgcatttcaaaagttggattaaaggg
ttgtaactgactacagcatggaaaaaaatagttcttttaattctttcaccttaaagcatattttatgtct
caaaagtataaaaaactttaatacaagtacatacatattatatatacacatacatatatatactatatat
ggatgaaacatattttaatgttgtttactttttaaatacttggttgatcttcaaggtaatagcgataca
attaaattttgttcagaaagtttgttttaaagtttattttaagcactatcgtaccaaatatttcatattt
cacattttatatgttgcacatagcctatacagtacctacatagtttttaaattattgtttaaaaaacaaa
acagctgttataaatgaatattatgtgtaattgtttcaaacatccattttctttgtgaacatattagtga
ttgaagtattttgacttttgagattgaatgtaaaatattttaaatttgggatcatcgcctgttctgaaaa
ctagatgcaccaaccgtatcattatttgtttgaggaaaaaaagaaatctgcattttaattcatgttggtc
aaagtcgaattactatctatttatcttatatcgtagatctgataaccctatctaaaagaaagtcacacgc
taaatgtattcttacatagtgcttgtatcgttgcatttgttttaatttgtggaaaagtattgtatctaac
ttgtattactttggtagtttcatctttatgtattattgatatttgtaattttctcaactataacaatgta
gttacgctacaacttgcctaaaacattcaaacttgttttctttttctgtttttttctttgttaattcat
ttaaactcattgaaaacatagtatacattactaaaaggtaaattatgggaatcactgaaatattttgta
gattaattgttgtaacattgtctttctttttttttcttttgtttcatgattttgattttaaaattattag
cacacaactattttcagccctttaataatggagcatcaaaaacatcacctgtaaccccaagcaaatatag
aagactgtatttttttactatgatatccattttccagaattgtgattacaatatgcaaagagtcataaata
tgccatttacaataaggaggaggcaaggcaaatgcatagatgtacaaatatatgtacaacagattttgct
ttttatttatttataatgtaattttatagaataattctgggatttgagaggatctaaaactatttttctg
tataaatattatttgccaaaagtttgtttatattcagaagtctgactatgatgaataaatcttaaatgct
ttgtttaattaaaaaacaaaaatcaccaatatccaagacatgaagatatcagttcaacaaatactgtagt
taagagactaactctccacttgtatgggaactacatttcactcttggttttcaggatataacagcacttc
accgaaatattctttcagccataccactggtaacatttctactaaatctttctgtaacacttaaagaatt
ccctcattcattaccttacagtgtaaacaggagtctaatttgtatcaatactatgttttggttgtaatat
tcagttcactcacccaatgtacaaccaatgaaataaaagaagcatttaaaaggaaaaaaaaaaaaaaaaa
aaaaaaaaaaa

MUTAGENESIS:

> MBNL1 218 PM
tcagcagaaacggaatggaatgccaagaatctgcattgagaataactaaacattgttactgtacatacta
tcctgtttcctcctcaatagaattgccacaaactgcatgctaaataaagatgtagttcttctggacagac
cacaactctaagaagctagtgctgctatctcatatatgagtattaaatatggtatgcttagtatattcca
acctaagatagttaactacctgagaccagctgtgatgtttaaagacataaaggataaagtttacttttaa
agggtttctaaacatagtttctgtcctaggaatattgtcttatctccataactatagctgatgcagaaag
tccagccagtttactcatttcgattcagaatatttcaaatttagcaataaacaattagcattagttaaaa
aagaaacatattccaagggcaggttcgattctagctctaattactgtcatgtcatttacccactggatca
aagggtatgtttcacttcttgacaatataaatgctgcagcaaagatgagaggtgaagtaaaaccgatacc
tgtcctgcaggtctaaaatttACaTGgTTaGAtcaagcacaagtactggggacacatcaaagtgtggtgt
ttggtttgcctggagatgccacgttgaatcatgtgattctagattaacattaaatagattgaaaaagaaa
ctttgcacggtatgagcttcataccccaccaaacaaagtcttgaaggtattattttacaagtatattttt
aaagttgttttataagagagactttgtagaagtgcctagattttgccagacttcatccagcttgacaaga
ttgagaggcccatgccaacagtctaatctaagagattagtctttcaaactcaccatccagttgcctgtta
cagaataactcttcttaactaaaaacctagtcaaacaaggaagctgtaggtgaggagatctgtataatat
tctaatttaagtaagtttgagtttagtcactgcaaatttgactgtgactttaatctaaattactatgtaa
acaaaaagtagatagtttcactttttaaaaaatccattactgttttgcatttcaaaagttggattaaagg
gttgtaactgactacagcatggaaaaaaatagttcttttaattctttcaccttaaagcatattttatgtc
tcaaaagtataaaaaactttaatacaagtacatacatattatatatacacatacatatatatactatata
tggatgaaacatattttaatgttgtttactttttaaatacttggttgatcttcaaggtaatagcgatac
aattaaattttgttcagaaagtttgtttACaTgGttaGAtCaagcacAatcgtaccaaatatttcatatt
tcacattttatatgttgcacatagcctatacagtacctacatagtttttaaattattgtttaaaaaacaa
aacagctgttataaatgaatattatgtgtaattgtttcaaacatccattttctttgtgaacatattagtg
attgaagtattttgacttttgagattgaatgtaaaatattttaaatttgggatcatcgcctgttctgaaa
actagatgcaccaaccgtatcattatttgtttgaggaaaaaaagaaatctgcattttaattcatgttggt
caaagtcgaattactatctatttatcttatatcgtagatctgataaccctatctaaaagaaagtcacacg
ctaaatgtattcttacatagtgcttgtatcgttgcatttgttttaatttgtggaaaagtattgtatctaa
cttgtattactttggtagtttcatctttatgtattattgatatttgtaattttctcaactataacaatgt
agttacgctacaacttgcctaaaacattcaaacttgttttctttttctgtttttttctttgttaattca
tttaaactcattgaaaacatagtatacattactaaaaggtaaattatgggaatcactgaaatattttgt
agattaattgttgtaacattgtctttctttttttttcttttgtttcatgattttgaACAtGGTTaGATCAa
gcacaAaactattttcagccctttaataatggagcatcaaaaacatcacctgtaaccccaagcaaatata

gaagactgtatttttttactatgatatccattttccagaattgtgattacaatatgcaaagagtcataaat
atgccatttacaataaggaggaggcaaggcaaatgcatagatgtacaaatatatgtacaacagattttgc
ttttatttatttataatgtaattttatagaataattctgggatttgagaggatctaaaactatttttct
gtataaatattatttgccaaaagtttgtttatattcagaagtctgactatgatgaataaatcttaaatgc
tttgtttaattaaaaaacaaaaatcaccaatatccaagacatgaagatatcagttcaacaaatactgtag
ttaagagactaactctccacttgtatgggaactacatttcactcttggttttcaggatataacagcactt
caccgaaatattctttcagccataccactggtaacatttctactaaatctttctgtaacacttaaagaat
tccctcattcattaccttacagtgtaaacaggagtctaatttgtatcaatactatgttttggttgtaata
ttcagttcactcacccaatgtacaaccaatgaaataaaagaagcatttaaaaggaaaaaaaaaaaaaaa
aaaaaaaaaaaa

> MBNL1 23b PM
tcagcagaaacggaatggaatgccaagaatctgcattgagaataactaaacattgttactgtacatacta
tcctgtttcctcctcaatagaattgccacaaactgcatgctaaataaagatgtagttcttctggacagac
cacaactctaagaagctagtgctgctatctcatatatgagtattaaatatggtatgcttagtatattcca
acctaagatagttaactacctgagaccagctgtgatgtttaaagacataaaggataaagtttacttttaa
agggtttctaaacatagtttctgtcctaggaatattgtcttatctccataactatagctgatgcagaaag
tccagccagtttactcatttcgattcagaatatttcaaatttagcaataaacaattagcattagttaaaa
aagaaacatattccaagggcaggttcgattctagctctaattactgtcatgtcatttacccactggatca
aagggtatgtttcacttcttgacaatataaatgctgcagcaaagatgagaggtgaagtaaaaccgatacc
tgtcctgcaggtctaaaatttgaatggaaattcaagcacaagtactggggacacatcaaagtgtggtgtt
tggtttgcctggagaGGTAaTCCCTGGCAatgtgattctagattaacattaaatagattgaaaaagaaac
tttgcacggtatgagcttcataccccaccaaacaaagtcttgaaggtattattttacaagtatattttta
aagttgttttataagagagactttgtagaagtgcctagattttgccagacttcatccagcttgacaagat
tgagaggcccatgccaacagtctaatctaagagattagtctttcaaactcaccatccagttgcctgttac
agaataactcttcttaactaaaaacctagtcaaacaaggaagctgtaggtgaggagatctgtataatatt
ctaatttaagtaagtttgagtttagtcactgcaaatttgactgtgactttaatctaaattactatgtaaa
caaaaagtagatagtttcacttttaaaaaatccattactgttttgcatttcaaaagttggattaaaggg
ttgtaactgactacagcatggaaaaaaatagttcttttaattctttcaccttaaagcatattttatgtct
caaaagtataaaaaactttaatacaagtacatacatattatatatacacatacatatatatactatatat
ggatgaaacatattttaatgttgtttactttttaaatacttggttgatcttcaaggtaatagcgataca
attaaattttgttcagaaagtttgttttaaagtttattttaagcactatcgtaccaaatatttcatattt
cacattttatatgttgcacatagcctatacagtacctacatagtttttaaattattgtttaaaaaacaaa
acagctgttataaatgaatattatgtgtaattgtttcaaacatccattttctttgtgaacatattagtga
ttgaagtattttgacttttgagattgaatgtaaaatattttaaatttgggatcatcgcctgttctgaaaa
ctagatgcaccaaccgtatcattatttgtttgaggaaaaaaagaaatctgcattttaattcatgttggtc
aaagtcgaattactatctatttatcttatatcgtagatctgataaccctatctaaaagaaagtcacacgc
taaatgtattcttacatagtgcttgtatcgttgcatttgttttaatttgtggaaaagtattgtatctaac
ttgtattactttggtagtttcatctttatgtattattgatatttgtaattttctcaactataacaatgta
gttacgctacaacttgcctaaaacattcaaacttgtttcttttttctgtttttttctttgttaattcat
ttaaactcattgaaaacatagtatacattactaaaaggtaaattatgggaatcactgaaatattttgta
gattaattgttgtaacattgtctttctttttttttcttttgtttcatgattttgattttaaaattattag
cacacaactattttcagccctttaataatggagcatcaaaaacatcacctgtaaccccaagcaaatatag
aagactgtatttttttactatgatatccattttccagaattgtgattacaatatgcaaagagtcataaata
tgccatttacaataaggaggaggcaaggcaaatgcatagatgtacaaatatatgtacaacagattttgct
tttatttatttataatgtaattttatagaataattctgggatttgagaggatctaaaactatttttctg
tataaatattatttgccaaaagtttgtttatattcagaagtctgactatgatgaataaatcttaaatgct
ttgtttaattaaaaaacaaaaatcaccaatatccaagacatgaagatatcagttcaacaaatactgtagt
taagagactaactctccacttgtatgggaactacatttcactcttggttttcaggatataacagcacttc
accgaaatattctttcagccataccactggtaacatttctactaaatctttctgtaacacttaaagaatt
ccctcattcattaccttacagtgtaaacaggagtctaatttgtatcaatactatgttttggttgtaatat
tcagttcactcacccaatgtacaaccaatgaaataaaagaagcatttaaaaggaaaaaaaaaaaaaaa
aaaaaaaaaaa
